# Structure–Property Relationships in Auxetic Liquid Crystal Elastomers—The Effect of Spacer Length

**DOI:** 10.3390/polym16141957

**Published:** 2024-07-09

**Authors:** Stuart R. Berrow, Thomas Raistrick, Richard J. Mandle, Helen F. Gleeson

**Affiliations:** 1 School of Physics and Astronomy, University of Leeds, Leeds LS2 9JT, UK; phytr@leeds.ac.uk (T.R.); r.mandle@leeds.ac.uk (R.J.M.); h.f.gleeson@leeds.ac.uk (H.F.G.); 2 School of Chemistry, University of Leeds, Leeds LS2 9JT, UK

**Keywords:** liquid crystal elastomer, auxetic, spacer, structure-property relationships, mechanical metamaterials

## Abstract

Auxetics are materials displaying a negative Poisson’s ratio, i.e., getting thicker in one or both transverse axes when subject to strain. In 2018, liquid crystal elastomers (LCEs) displaying auxetic behaviour, achieved via a biaxial reorientation, were first reported. Studies have since focused on determining the physics underpinning the auxetic response, with investigations into structure–property relationships within these systems so far overlooked. Herein, we report the first structure–property relationships in auxetic LCEs, examining the effect of changes to the length of the spacer chain. We demonstrate that for LCEs with between six and four carbons in the spacer, an auxetic response is observed, with the threshold strain required to achieve this response varying from 56% (six carbon spacers) to 81% (four carbon spacers). We also demonstrate that Poisson’s ratios as low as −1.3 can be achieved. Further, we report that the LCEs display smectic phases with spacers of seven or more carbons; the resulting internal constraints cause low strains at failure, preventing an auxetic response. We also investigate the dependence of the auxetic threshold on the dynamics of the samples, finding that when accounting for the glass transition temperature of the LCEs, the auxetic thresholds converge around 56%, regardless of spacer length.

## 1. Introduction

**Figure 1:**
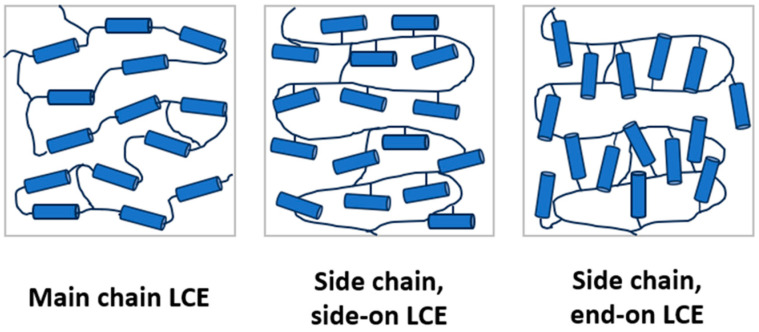
Schematics showing the potential architectures for LCEs, with the blue rods representing the mesogenic units and lines representing polymer chains.

Liquid crystal elastomers (LCEs) are lightly cross-linked polymers, which combine the properties of elastomers (i.e., elasticity) with those of liquid crystals (i.e., self-organisation and anisotropy) [1,2]. This combination of properties is achieved by incorporating anisotropic units, known as mesogens, into the polymer structure. The integration of mesogens into the LCE can be achieved either by incorporation directly into the polymer backbone to yield main chain LCEs (MCLCEs) or by attaching the mesogens to the backbone via a flexible group (known as a spacer) to yield side chain LCEs (SCLCEs) (Figure 1) [1]. 

**Figure 2:**
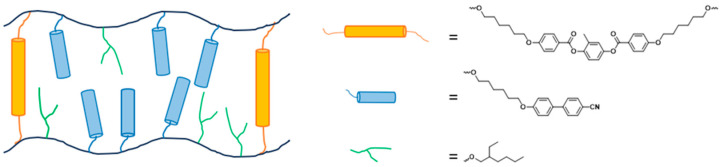
A schematic representation of the LCE reported by Mistry et al. [8] displaying auxetic behaviour.

The mesogens in the polymer structure have an affinity to self-organise, leading to the LCEs exhibiting long-range order [2]. This, in turn, is coupled to the macroscopic shape of the LCE [2]. This coupling of liquid crystalline order and macroscopic polymer properties gives rise to some well-known interesting behaviours, including stimuli-responsiveness, stress-optical coupling and enhanced damping [3,4,5,6,7]. Much of the literature focuses on investigations into MCLCEs, as the interesting actuating behaviours they exhibit are generally more pronounced than for SCLCEs. In 2018, Mistry et al. reported the observation of a novel behaviour for LCEs, the ability for an LCE to display an auxetic response (i.e., a negative Poisson’s ratio) when subject to strain perpendicular to the nematic director [8]. It is proposed that the auxetic response in LCEs could be useful for application in biomedical devices, impact resistance or delamination resistance [8,9,10]. The LCE in question has an acrylate backbone and can be described as a predominantly SCLCE, showing a small quantity of MCLCE characteristics due to the use of the mesogenic cross-linker (Figure 2). All of the auxetic LCEs reported to date display this predominantly SCLCE nature. 

The LCE reported by Mistry et al. is auxetic along an axis perpendicular to both the liquid crystal director and the strain direction, beyond a threshold strain value [8]. The response is facilitated by the anisotropic nature of LCEs, which allows for a negative Poisson’s ratio in one transverse axis, provided the other transverse axis has a positive Poisson’s ratio, thereby conserving volume [8,11]. Whilst the notion that liquid crystal polymers could display auxetic behaviour had been suggested many years earlier, this was the first example of an LCE in which an auxetic response had been directly observed [12,13]. Significantly, the LCE was proven to be non-porous, unlike all other synthetic auxetics previously reported, marking the first example of a synthetic, non-porous, molecular auxetic material [8]. 

The physical phenomena underpinning the auxetic response in the auxetic LCEs reported by Mistry et al. have been deduced from studies within our research group [8,11,14]. The auxetic LCEs were seen to deform via the so-called ‘mechanical Fréedericksz transition’ (MFT), as opposed to the more commonly observed semi-soft elastic (SSE) response [8]. LCEs deforming by an SSE mechanism experience a continuous, in-plane rotation of the director to align with the applied strain [11,15,16,17]. On the other hand, the MFT mechanism has recently been shown to be a continuous biaxial reorientation of the director with a growing proportion of mesogens orientated along both the strain axis and the transverse axis perpendicular to the director [11]. 

In addition to the observation of an MFT deformation, a significant change in the liquid crystal order is observed in the auxetic LCEs. Raistrick et al. proposed that the auxetic response was connected to an out-of-plane rotation of the director, resulting in the emergence of biaxial order in the sample [11]. The strain-induced biaxiality was confirmed by Wang et al., who used conoscopy on homeotropically aligned LCEs to conclusively show the emergence of biaxial order in the LCE upon the application of strain perpendicular to the nematic director, underpinning the auxetic response [14].

Perhaps one of the most exciting aspects of an LCE that has an auxetic response is the potential for tuning the mechanical behaviour through chemical modifications. So far, this opportunity has been largely neglected, with the focus instead on understanding the physical basis of the auxetic response, thereby allowing the deduction of some theory-based design rules. Those studies concentrated on understanding how out-of-plane deformation could occur and therefore only considered two very closely related materials [8,11,14]. In this paper, we report the first steps in exploring both the robustness of the auxetic response to changes in the LCE design and structure–property correlations of the auxetic behaviour by changing the spacer length of the monofunctional sidechain unit.

## 2. Materials and Methods

### 2.1. Monomer Synthesis

The series of monomers used in this work will be henceforth referred to as the AnOCB series, where the n in the monomer abbreviation denotes the number of methylene units in the spacer chain. For example, A3OCB is the monomer with 3 methylene units in the spacer (3-(4-Cyano-biphenyl-4′-yloxy)propyl acrylate). Generalized synthetic procedures and experimental data for these syntheses are detailed in the Supplementary Information.

### 2.2. Elastomer Mould Fabrication

The LCEs were synthesized in bespoke alignment moulds, which were made in accordance with the previous literature [8,11,14]. A glass microscope slide (7.5 cm × 2.5 cm × 1 mm) and a Melinex^®^ ST725 substrate (7 cm × 2.5 cm × 250 µm) (DuPont Teijin Films, Redcar, UK) were spin-coated on one surface with an aqueous 0.5 wt% polyvinyl alcohol (PVA) solution, which was uniaxially rubbed with a bespoke rubbing machine after drying the substrates at 50 °C for 15 min. These two substrates were then adhered, via Melinex^®^ 401 spacers (7.5 cm × 0.2 cm × 100 µm) (DuPont Teijin Films, Redcar, UK) and UVS-91 adhesive (Edmund Optics, York, UK), so that the PVA-rubbed surfaces were the inner surfaces of the constructed cell, and the rubbing directions yielded antiparallel planar alignment along the width of the mould. The adhesive was then cured by irradiation under 350 nm (2.5 Wcm^−2^) at 50 °C for 10 min, to yield the constructed LCE mould with a gap thickness of ~100 µm. The moulds produced are highly uniform, consistently yielding samples with the desired alignment and thickness variations in the region of 5%, as reported in previous work [18]. 

### 2.3. Liquid Crystal Elastomer Synthesis (Planar Alignment)

**Figure 3:**
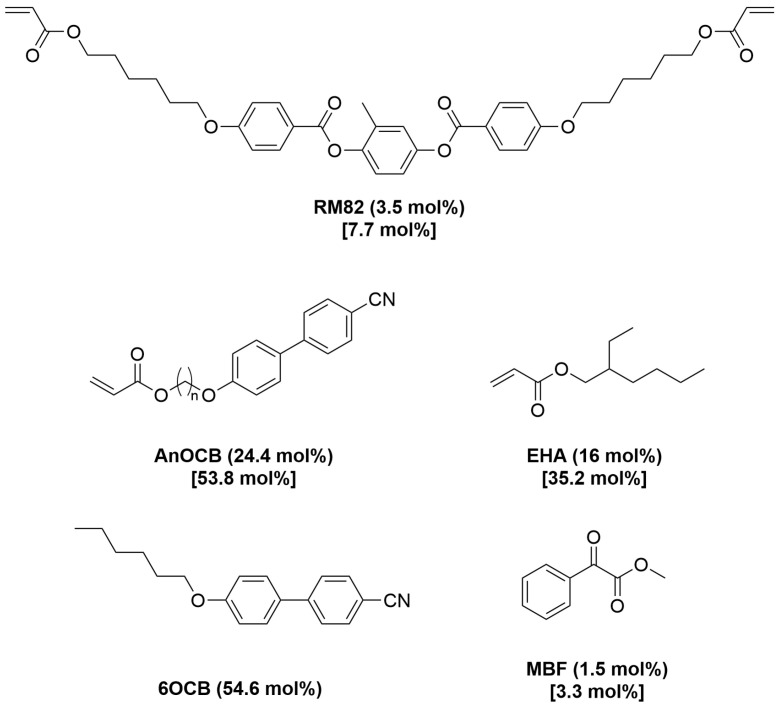
The composition of the LCE precursor mixtures, where the AnOCB monomer is varied to give the appropriate spacer length. The values in brackets give the composition of the LCE precursor mixture, and the values in square brackets display the composition of the final LCE.

The LCEs synthesized in this work were made via a method adapted from previous work within the group [8,14,19]. A generalised procedure is given below, and the generalised monomer mixture is displayed in Figure 3. Table 1 details the nomenclature used for the LCEs fabricated in this way. LCE systems are named AN0n, where n refers to the number of methylene units in the acrylate-terminated alkoxycyanobiphenyl, i.e., AN03 uses the acrylate monomer A3OCB, which has three methylene units.

In a typical procedure, RM82 (3.5 mol%), the cyanobiphenyl monomer AnOCB (24.4 mol%), and 6OCB (54.6 mol%) were heated to 120 °C with stirring until a homogeneous isotropic phase was obtained. The mixture was cooled to 50 °C, followed by the addition of EHA (16 mol%) and MBF (1.5 mol%), and stirred for 5 min, again ensuring a homogeneous, completely isotropic material was obtained. The mixture was then filled into a mould at 50 °C via pipette, before being cooled to room temperature and allowed to stand for 20 min. The samples were then cured under 350 nm (2.5 Wcm^−2^) irradiation for 2 h, to yield a fully cured sample as evidenced in previous work [18]. After curing, the samples were removed from the moulds (using a small amount of isopropanol if necessary to aid delamination from the substrates), and left to stand in a solution of dichloromethane (DCM)–isopropanol (30:70) overnight to remove the non-reactive 6OCB. The samples were then allowed to dry under ambient conditions for 5 h, to yield the final LCE films.

### 2.4. Material Characterisation

Full experimental information for the characterization carried out in this work can be found in the Supplementary Information. This includes structural analysis of synthesized compounds, thermal analysis of LCE precursors and final LCEs via differential scanning calorimetry, optical microscopy of the precursors and LCEs, phase determination for the LCEs by X-ray scattering, order parameter measurements for the LCEs via Raman spectroscopy and mechanical analysis to determine the auxetic threshold of the LCEs. 

## 3. Results

The LCEs were characterized extensively, and significant results are summarized in Table 2. These results will be discussed in detail throughout this work.

### 3.1. Liquid Crystal Elastomer Production

To examine the effect of spacer length on LCE properties, care was taken to ensure that the molar ratio of the monomers in the LCE mixture was consistent throughout, with the LCEs having the composition depicted in Figure 3. This composition is based on the composition of the auxetic LCE reported by Wang et al., which, in this work, is known as AN06 and differs from the Mistry et al. LCE slightly in terms of cross-link density; AN06 has 7.7 mol% of crosslinker (RM82) compared to ~17 mol% in Mistry et al. [8,14], and its auxetic threshold is at lower strains and further away from the point of sample failure than for the Mistry et al. LCE [8,14]. It is of note that 6OCB is used to template the nematic phase in the unpolymerized mixtures for all systems. It is an unreactive component, included to ensure that the precursor mixture is in a nematic phase, allowing high-quality monodomain alignment of the final LCE. The 6OCB is removed from the final LCE resulting in a different composition from the unpolymerized mixture (Figure 3).

**Figure 4:**
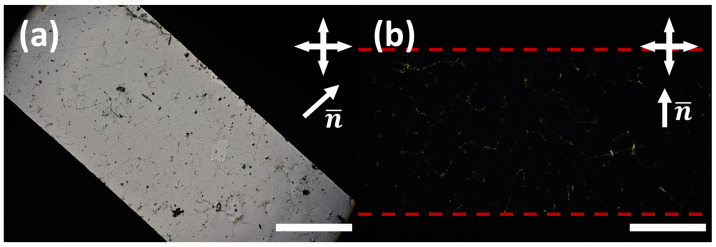
Polarised optical microscopy images to show the planar alignment achieved in the LCEs, showing the bright state (**a**) and dark state (**b**) upon 45° rotation (this example was taken from an AN08 LCE), using a sample of 20 mm × 2 mm. The scale bar represents 1 mm. In (**b**), the dotted red lines are added to denote the edges of the sample.

In this work, macroscopic, high-quality LCEs, usually with planar alignment, were targeted, as well-defined liquid crystal alignment is imperative for the auxetic response to be observed [7,8]. To achieve this, polymerisation of the mixture whilst in the nematic phase is crucial. When examined by differential scanning calorimetry (DSC), the LCE precursor mixtures all display enantiotropic nematic phases, with clearing temperatures varying from 34–45 °C (Figures S1 and S2). All the precursor mixtures can therefore be polymerized at room temperature in the nematic phase in a mould with the desired surface alignment conditions, as is the case with the previously reported auxetic LCEs [8,14]. In all cases, LCEs exhibiting excellent planar alignment are routinely achieved, as confirmed by colour inversion of planar samples when the LCE films are rotated by 45 °C under crossed polarisers (Figure 4).

Having successfully fabricated LCEs with planar alignment, confirmation of the liquid crystal phase exhibited by the LCEs was sought [8,14]. Small-angle X-ray scattering (SAXS) and wide-angle X-ray scattering (WAXS) experiments were conducted on samples of each elastomer to investigate this. The one-dimensional (Figure S3) and two-dimensional (Figures S4 and S5) SAXS and WAXS data confirm that for spacer lengths of fewer than seven methylene units, the LCEs display a nematic phase. This is evidenced by the diffuse peaks in both SAXS and WAXS data and the lack of any pronounced reflections at small angles.

Conversely, for the LCEs fabricated with monomers containing spacers of seven or more methylene units, the presence of a relatively sharp (001) Bragg peak observed in the SAXS data at around q = 1.5 nm^−1^ suggests a smectic phase. In the 2D WAXS data (Figure S5), the sharp small-angle peaks are orthogonal to the diffuse scattering resulting from side-to-side intermolecular spacings. We therefore attribute the phase to be smectic A. These sharp reflections correspond to layer spacings of 38.5 Ǻ, 40.5 Ǻ and 40.5 Ǻ for the AN07, AN08 and AN09 LCEs, respectively. These layer spacings are comparable to the average end-to-end length of RM82 (38.4 Ǻ, calculated as detailed in Supplementary Information), rather than the monofunctional monomer lengths (23.2 Ǻ, 29.1 Ǻ and 23.8 Ǻ for A7OCB, A8OCB and A9OCB, respectively), which suggests that the layer spacings are dictated by RM82, which adopts a slightly strained conformation and not significantly influenced by the choice of AnOCB monomer. All AnOCB monomers have average end-to-end lengths of 21.2–29.1 Ǻ, which suggests the AnOCB side groups adopt a partially interdigitated structure within the smectic layers.

These observations agree with the literature, which shows that for end-on-side chain liquid crystal polymers and elastomers, increased spacer lengths commonly yield smectic phases [20,21,22,23,24,25]. However, given the nematic templating employed in the LCE mixture, we find the formation of smectic phases to be interesting, as the curing of the precursor mixture in the nematic phase might be expected to yield an LCE with a defined nematic order. A detailed exploration of phase templating is beyond the scope of this article and will be reported at a later date.

### 3.2. Thermal Analysis of LCEs

The presence of any thermally induced phase transitions within the LCEs was examined by DSC. Key results are displayed in Table 2, and an example thermogram is given for each LCE in the Supplementary Information (Figures S6–S12). All LCEs display a glass transition temperature (*T*_g_), indicated by the step change observed in the DSC thermograms, below or approaching room temperature. The results indicate that as the number of methylene units in the spacer chain increases, the *T*_g_ of the material decreases, a consequence of the increased flexibility of the spacers as more carbon atoms are added, consistent with the literature for side-chain liquid crystal polymers and elastomers [21,26,27,28,29,30]. When examined as a function of spacer length (Figure S13), there is no apparent presence of odd–even effects in *T*_g_, which is, again, consistent with the literature in which, at most, only weak odd–even effects are observed in *T*_g_ [21,26,27,28,29,30].

In all cases, the DSC analysis of the LCEs shows no evidence of any further phase transitions prior to thermal degradation, consistent with the behaviour of the previously reported auxetic LCEs [8,11,14]. This behaviour is consistent with that of more highly cross-linked liquid crystal networks (LCNs) as opposed to LCEs, an interesting observation given the relatively low cross-link density of the LCEs (7.7 mol%) [1]. However, SAXS analysis of the smectic A LCEs (AN07, AN08 and AN09) as a function of temperature showed some evidence of a decrease in smectic order as temperature increases, though the layer spacing appears to change little as a function of temperature (Figures S16–S21). The reduction in smectic order is most notable at 110 °C, which suggests the possible presence of a transition from the smectic A phase in these LCEs. The lack of any evidence of this transition in DSC and the observations made by SAXS suggest that if this is indeed a phase transition, it is weak first-order or second-order in nature.

In addition to the lack of an apparent *T*_c_ in DSC data, the thermally induced shape changes seen in both the nematic and smectic-A LCEs (Figure S14) show no evidence of the significant actuation expected at or near a phase change such as *T*_c_. This is further evidence of thermal behaviour more typical of LCNs. Furthermore, there is no significant difference in thermal shape change between the LCEs showing a nematic character and a smectic character. This is unexpected based on previous literature findings, in which smectic LCEs show an increase in length with increased temperature, before a sharp reduction in length upon transition to the isotropic phase; this is the opposite of the steady decrease in elongation with increasing temperature observed for nematic LCEs, with, again, a sharp reduction in length upon transition into the isotropic phase [31].

The work from Raistrick et al. suggests that a high clearing temperature (*T*_c_) is an important factor for LCE being able to display an auxetic response [11]. They suggest that when strained at temperatures far below *T*_c_, as is the case in the auxetic LCEs previously reported, the biaxial stiffness of the material is expected to be significantly lower than the uniaxial stiffness [11]. This allows biaxiality to dominate, which in turn leads to the out-of-plane mesogen reorientation to yield the auxetic response. The lack of any apparent *T*_c_ in these LCEs suggests that if strained at room temperature, they will satisfy this criterion and hence display an auxetic response.

### 3.3. Mechanical Analysis

A key motivation of this work was to determine the dependence of an auxetic response (negative Poisson’s ratio) in the LCE films on the spacer length of the mesogen, so the mechanical analysis of the materials focuses on this feature. The presence of an auxetic response in the LCEs was examined with a bespoke experimental setup, the full specifications of which have been described previously [8]. In brief, the sample (of approximate dimensions of 20 mm × 2 mm × 100 µm) is loaded between two actuators, and the initial actuator separation is set to a distance that is sufficient to remove any slack in the sample. The samples were then subject to strain steps of 0.5 mm (the smallest possible step on the bespoke apparatus, allowing the highest-resolution data) at 10 min intervals until sample failure. During this process, images of the sample both through optical microscopy and polarized optical microscopy were taken at each strain step.

**Figure 5:**
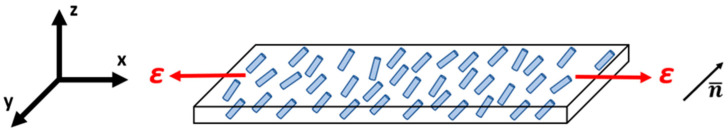
A schematic representation of the initial nematic director orientation ($\bar{n}$) relative to the strain axis (ε) of the auxetic LCEs for the mechanical analyses conducted in this work. The mesogenic units (both side-chain and cross-linker) are shown schematically as blue cylinders with their average direction (the director) indicated.

**Figure 6:**
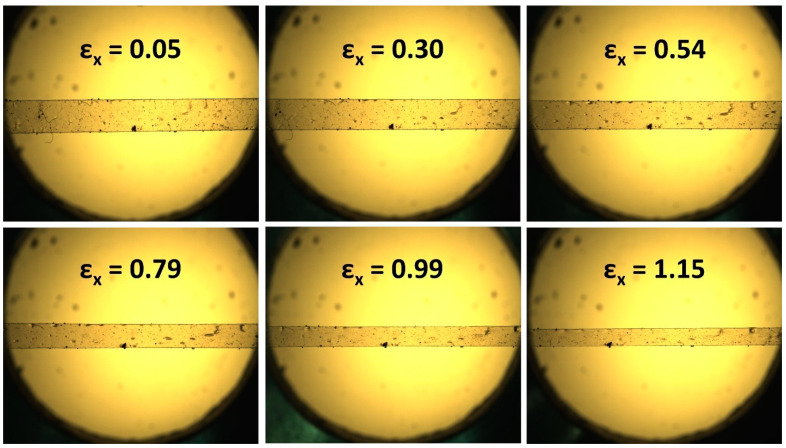
Example optical microscopy images obtained for the mechanical experiments undertaken in this work, showing the observation of the x-y plane as a function of applied strain. In this case, the images show an AN05 sample, being examined at room temperature (22 °C). The auxetic threshold is determined from analysis of the full set of images, but a significant reduction in width can be seen at strains above 0.54, indicative of thickening of the sample in the z-direction.

A schematic representation of the mechanical analysis employed in this work is given in Figure 5. The images taken during this work examine the x-y plane of the sample, whereas the auxetic response is observed along the z-axis. Particle tracking is used to calculate the strain in both the x- and y-axes from the recorded images, and the z strain is then inferred based on the conservation of volume. The conservation of volume has been proven for auxetic LCEs in previous work, with this methodology providing results indistinguishable from those seen via direct observation of the auxetic plane [8,11,14]. The auxetic response can be visualized in the x-y plane as a significant decrease in the width of the sample, as depicted by the example images in Figure 6.

To determine the auxetic threshold for each LCE, Poisson’s ratio of the sample in the z-axis is calculated, using a method applied previously [14]. In brief, the strains obtained from the experimental images (such as those in Figure 6) are engineering strains, which are converted into true strains using $\varepsilon_{true}=\ln\left(\varepsilon_{engineering}+1\right)$. A polynomial is then fit to these data, and Poisson’s ratio (*ν*) is calculated from $\nu =-\left(d\varepsilon_{trans}/d\varepsilon_{expan}\right)$, i.e., the ratio of the relative deformation in the transverse direction of expansion to the relative expansion. In this work, the deformation in the z-axis is the transverse deformation ($d\varepsilon_{trans}$), and the strain applied in the x-axis is the relative expansion ($d\varepsilon_{expan}$). The point at which the Poisson’s ratio in the z-axis becomes negative is the auxetic threshold. It is noteworthy that the average Poisson’s ratio of the materials in both transverse dimensions is observed to be 0.5 throughout the experiment, consistent with that of many elastomeric materials [32].

**Figure 7:**
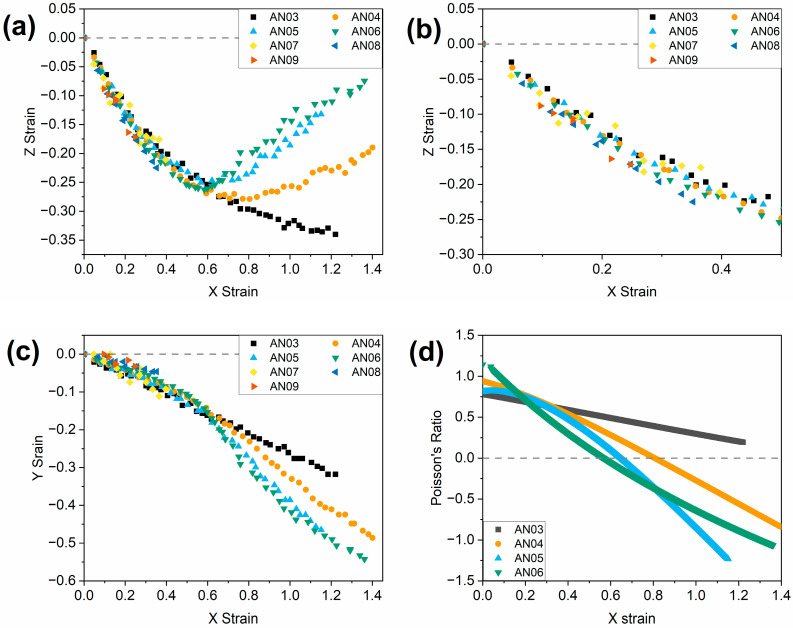
The effect of an applied x strain on (**a**) the observed z strain for the LCE samples, (**c**) the observed y strain in the LCE samples, and (**d**) the Poisson’s ratio in the z-dimension for the LCE samples, at 22 °C in all cases. Graph (**b**) is an enhanced area of graph (**a**), focusing on the region of 0–0.5 in x strain to aid visualisation of data.

In the first instance, the presence of an auxetic response in all LCEs was explored at room temperature (22 °C). Figure 7a,c show the result of an applied strain in the x-dimension on the strain observed in the z- and y-dimensions, respectively. Figure 7d displays the instantaneous Poisson’s ratio in the z-dimension for the LCEs, calculated as described, and as a function of the applied x-strain. For all LCEs, the samples are observed to undergo a thinning in the y-dimension throughout the whole experiment, upon the application of a strain in the x-dimension, as displayed in Figure 6. Upon the initial application of a strain in the x-dimension, all LCEs are also seen to undergo thinning in the z-dimension up to the point of the auxetic threshold, where one exists. Beyond this threshold, for AN04, AN05 and AN06, the LCEs are observed to become thicker in the z-dimension; Poisson’s ratio in the z-dimension becomes negative. This behaviour is analogous to that of the original auxetic LCE from Mistry et al. and confirms that these three LCEs have an auxetic response at 22 °C.

The strains required to reach the auxetic threshold for the AN04, AN05 and AN06 LCEs at room temperature are displayed in Table 2. The auxetic threshold of AN06 is in agreement with that previously reported for the same material [14]. The results suggest that as the number of methylene units in the spacer is decreased, the auxetic threshold strain increases. This increase is such that the AN03 elastomer does not display an auxetic response at room temperature prior to failure (at ~120% strain). These results confirm that the auxetic response of the LCEs at room temperature can be tailored by modifications in the chemical structure.

We now consider AN07, AN08 and AN09, which are found to not exhibit an auxetic response at room temperature; indeed, these LCEs fail at relatively low strains (<0.40 in all cases). This low strain at failure is attributed to strong internal constraints resulting from the smectic layers of these LCEs, which can result from crosslinks within the layers and/or the very high elastic and compression moduli seen in smectic LCEs [33,34,35]. In our case, the relatively low strains at failure result in samples being unable to reach an auxetic threshold, if one occurs, in the smectic-A LCEs.

The behaviour of smectic A LCEs when subject to the application of strain has drawn interest in the literature, but no consensus has been reached. Most commonly, a significant anisotropy is observed depending on the direction of applied strain relative to the director/smectic layer normal [36,37,38,39,40,41,42,43,44]. Upon strain parallel to the layer normal, after a threshold strain of around 5% is reached, the samples mechanically behave as an isotropic network with a Poisson’s ratio of 0.5 in both transverse axes [36,37,38,39,40,41,42,43,44]. In some cases, this coincides with the samples developing a cloudy texture [36,40], attributed to a reorientation of smectic layers consistent with a Helfrich–Hunault type transition, whereas in other cases, no such opaqueness is observed [42]. Conversely, when strained perpendicular to the layer normal (as is the case in our work), the width of the sample (i.e., parallel to the layer normal) remains unchanged, and in order to conserve volume, the sample thickness is significantly reduced, following a Poisson’s ratio of 1.0 [36,37,38,39,40,41,42,43,44]. This is commonly attributed to an absence of any reorientation event under strain. It is of note, however, that all of the examples in which this behaviour is observed are siloxane-based SCLCEs, as opposed to the acrylate-based networks studied in this work.

Other works have suggested different behaviours for smectic A LCEs when subject to strain. Beyer et al. reported that for smectic A MCLCEs, a significantly lower anisotropy in the mechanical behaviour is observed [45]. This difference is attributed to the main chain systems having a tendency to form folded chain structures, which in turn results in short-range correlations for the smectic layers compared to those of SCLCEs [45]. Thus, the MCLCEs behave more similarly to nematic LCEs. Stannarius et al. reported a smectic-A SCLCE in which they observed a significant change in smectic layer spacing under strain, attributed to low smectic layer compressibility, but also discussed the possibility of strain-induced tilt [34]. A similar strain-induced tilt was proposed by Stenull and Lubensky in 2005 [46], indicative of a transition from uniaxial smectic A structure to a biaxial smectic C. It is noteworthy that the system in which this behaviour is observed by Stannarius et al. is a siloxane terpolymer, in which there are 2.7 times more non-mesogenic dimethyl siloxane repeat units than there are mesogenic repeat units [34]. It could be argued that as the materials studied in this work contain 35.2 mol% of the non-mesogenic monomer (EHA), the ‘diluted systems’ studied by Stannarius are perhaps a more appropriate means of comparison to the systems detailed here than a ‘fully substituted’ LCE.

**Figure 8:**
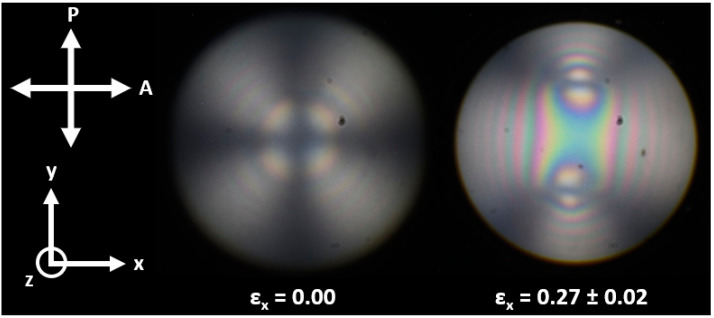
Conoscopic figures for a homeotropically aligned smectic LCE. The initial director and normal layers are aligned along the z-axis, strain has been applied along the x-axis, and the polarizer and analyser are aligned in the x- and y-axes, respectively. Two melatopes are clearly observable at a strain (ε_x_) of 0.27, indicative of biaxiality.

From the literature findings discussed, it is unclear as to whether one may expect the smectic A LCEs in this work to be capable of undergoing the reorientation required to create the biaxiality needed to see an auxetic response. To investigate the emergence of biaxiality within the smectic A LCEs upon the application of strain, conoscopy experiments were undertaken on homeotropic samples of the smectic LCEs. In the unstrained state, the conoscopic figure for the smectic LCEs shows a ‘Maltese cross’ texture, characteristic of a uniaxially aligned material (Figure 8). Upon the application of uniaxial strain in the x-direction, the conoscopic figure shows two melatopes, indicative of the emergence of multiple optical axes, and thus characteristic of a biaxial system (illustrated in Figure 8 at a strain of 0.27 where the melatopes are clearly observable). These observations agree with those for the nematic LCE AN06 reported by Wang et al. [14] and confirm the emergence of biaxiality with the smectic LCEs. Qualitative analysis of the conoscopic figures suggests a similar degree of biaxiality for AN07 and the AN06 LCEs reported by Wang et al. [14] (similar separation of the melatopes occurs at similar strains). However, the conoscopy figures could yield comparable results for the strain-induced smectic-A-to-smectic-C transition suggested by Stenull and Lubensky [46]. We believe this raises an interesting question. Is the emergence of biaxiality reported here due to a reorientation effect analogous to that seen in the nematic LCEs, or is this evidence of a strain-induced smectic-A-to-smectic-C transition? This will be the subject of future work.

The thermal analysis discussed earlier detailed a lack of any liquid crystal phase transitions in the nematic LCEs prior to thermal degradation. An interesting area of investigation for the smectic LCEs AN07, AN08 and AN09 would be the potential for a material in which the auxetic response could be turned ‘on or off’, by inducing a transition into the nematic phase in the LCE. Whilst this is not a possibility with the LCEs detailed in this work, this is an area of investigation we will pursue further in future work.

Whilst the impact of liquid crystal phase transitions on the LCEs cannot be investigated, the effect of proximity to the *T*_g_ of the LCEs has been examined. The observation that increasing the spacer length in the LCEs leads to a reduction in *T*_g_ suggests that, when examined at the same temperature (22 °C in this case), different polymer dynamics are likely being probed. We were therefore interested to understand if the change in auxetic threshold observed as spacer length is varied is dominated by the change in chemical structure or by a change in dynamics resulting from the change in *T*_g_.

To assess this hypothesis, we took the room temperature behaviour of the original auxetic LCE, in this case, AN06, to be the marker against which the samples were measured. With this in mind, a ‘reduced temperature’ was calculated, at which to conduct experiments where the differences in glass transition had been accounted for, as shown in Equation (1). This was calculated as the fraction above *T*_g_ at which the AN06 samples were analysed. We then used this reduced temperature to calculate the temperature at which the other LCE sample experiments should be conducted to mimic the dynamics of the AN06 experiments. These temperatures are recorded in Table 2.

(1) $$Reduced\;temperature=\frac{T_{experiment}}{T_{g}}=\frac{295.15\;K}{279.15\;K}$$

**Figure 9:**
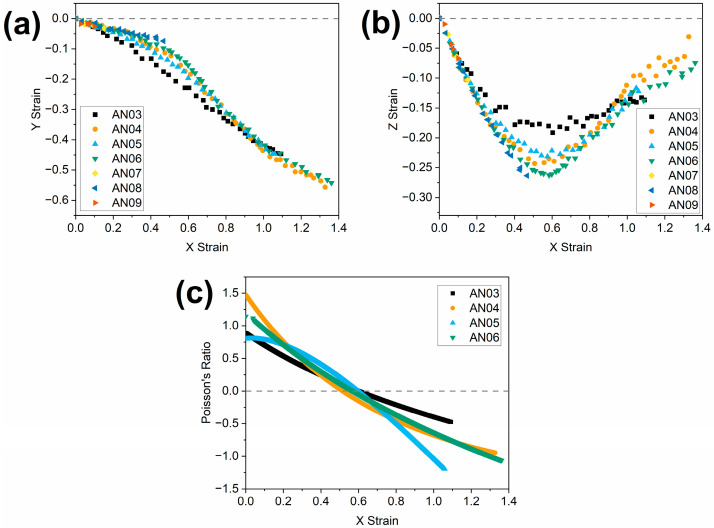
The effect of an applied X strain on (**a**) the observed Y strain for the LCE samples, (**b**) the observed Z strain in the LCE samples and (**c**) Poisson’s ratio in the Z dimension, at equivalent reduced temperatures (Table 2).

When the samples are examined at the appropriate experimental temperatures detailed in Table 2, the results (Figure 9) show some notable differences compared to the results obtained at room temperature (Figure 7). The nematic LCEs AN03, AN04, AN05 and AN06 exhibit auxetic responses with comparable auxetic thresholds (0.52–0.60) (Table 2). This convergence of auxetic thresholds suggests that the auxetic threshold of the nematic LCEs, and indeed therefore the presence of an auxetic response, is governed by proximity to *T*_g_, and thus the polymer dynamics being probed. It is, however, of note that the AN03 LCE shows a less negative Poisson’s ratio (−0.48) (and thus a less pronounced auxetic response) than the other LCEs (−0.95 to −1.20), suggesting that changes in the chemical structure may impact the magnitude of auxetic behaviour. In this case, we attribute this reduced auxetic response to a significant reduction in the flexibility of the spacer chain for the AN03 LCE. We suggest this has a two-fold effect. Firstly, the side chain itself is shorter, and thereby less likely to cause significant out-of-plane deformation as required for the auxetic response. Secondly, the AN03 spacer is significantly less flexible than that of the other LCEs, leading to more impedance to the out-of-plane mesogen reorientation, and thus a less pronounced response. When examined at the same reduced temperature as for the nematic LCEs, the AN07, AN08 and AN09 LCEs, which exhibit smectic ordering, still do not present an auxetic response, which is, again, attributed to the low strain at failure.

## 4. Discussion

When considered as a whole, these results present compelling information to further our understanding of the auxetic response in LCEs. We have demonstrated that the auxetic threshold of the LCE at room temperature can be tailored by changes in the length of the spacer in the major component of the LCE, with threshold strain values ranging from 0.56 to 0.81 observed. The observation that a relatively simple change in chemical structure can have such an impact on the auxetic response firstly shows that the auxetic behaviour observed within our previous work is not unique to the material previously reported (AN06 in this work), and perhaps is a more general phenomenon for LCEs.

In addition to the observed change in the auxetic threshold, the obtained evidence also suggests that the magnitude of the auxetic response can be tailored to some extent by changing the spacer length, with the minimum Poisson’s ratios observed ranging from −0.84 (AN04) to −1.23 (AN05). These results lead us to believe that the range of auxetic thresholds and Poisson’s ratios that can be achieved from LCEs could be vast, and we intend to explore the capabilities of LCEs with further chemical modifications, for example, variations in the mesogenic unit, in future work.

An important finding in this work is that the change in the auxetic threshold is revealed to be dominated by the change in the *T*_g_, as shown by the convergence of the auxetic threshold (in the region of 0.52 to 0.60) when all LCEs are analysed at the same reduced temperature (1.06 × *T*_g_ (K)). This suggests that proximity to *T*_g_ is an important factor in the observation of an auxetic response and may go some way to explain why, thus far, auxetic behaviour has only been observed in LCEs with acrylate backbones. The majority of the LCE literature focuses on either MCLCEs, synthesised through click chemistry, or SCLCEs with siloxane backbones, both of which lead to samples with far lower *T*_g_ than those typically observed for acrylate-based polymers. We hypothesise, therefore, that backbone chemistries that inherently have higher *T*_g_ (such as acrylates and methacrylates) are more conducive to facilitating an auxetic response. We therefore also intend to examine the scope for different backbone chemistries to yield an auxetic response in future work.

In addition to the impacts on the auxetic response previously detailed, this work also showed the synthesis of smectic A LCEs, despite polymerisation being conducted in a nematic phase. The formation of smectic phases in polymers from non-smectogenic monomers is known and results from an increase in order within the system as polymerisation constrains the mesogens, reducing their opportunity to flow or reorientate. This is particularly true in side-chain systems containing longer spacers, as the increasing spacer promotes microphase separation of aliphatic tails (the spacer) and aromatic cores [23]. It is therefore not unrealistic to suggest that this increased ordering is the cause of the smectic phase formation in the AN07, AN08 and AN09 LCEs. However, one could argue that the nematic order of the precursor mixture would be expected to be retained after curing, as is commonly the case in the synthesis of LCEs/LCNs. We therefore intend to probe the formation of the smectic phase further in future work. Additionally, we believe that the mechanical behaviour of the smectic A samples detailed in this work warrants further investigation, which would be beyond the scope of this article.

## 5. Conclusions

In this work, we have incorporated a series of cyanobiphenyl acrylate monomers of varying spacer lengths into novel liquid crystal elastomers (LCEs) with formulations consistent with the auxetic LCE reported in the previous literature. When cured, all elastomers exhibit excellent planar alignment as confirmed by polarised optical microscopy. For spacer lengths of ≤6 methylene units, the LCEs exhibit a nematic phase. However, for spacers containing seven or more methylene units, a smectic A phase is instead observed, even though polymerisation was conducted in a templated nematic phase. Thermal analysis of the LCEs shows that the glass transition temperature is reduced with increasing spacer length, consistent with increased flexibility in the repeat units. There are, however, no further phase transitions prior to thermal degradation of the LCEs, a behaviour typically observed in more highly cross-linked liquid crystal networks.

Analysis of the auxetic response of the LCEs at room temperature indicates that three of the LCEs exhibit an auxetic response. The LCEs exhibiting smectic A characteristics (AN07, AN08 and AN09) do not show an auxetic response due to low strains at failure resulting from constraints imparted by the layered structure. However, conoscopic analysis of the smectic A LCEs shows a transition from a uniaxial arrangement in the unstrained state to a biaxial arrangement upon the application of low strains. This is consistent with the molecular deformations previously observed to underpin the auxetic response in the existing auxetic LCEs. These results therefore suggest that the smectic A LCEs may be capable of auxetic behaviour, though constraints imposed by the layered structure lead to low strain at failure, and hence, no observed auxetic response.

For the nematic LCEs, reducing the spacer length of the cyanobiphenyl monomer leads to an increase in the auxetic threshold strain, at room temperature, with spacer lengths of four, five and six methylene units leading to auxetic thresholds of 0.57, 0.65 and 0.81, respectively. This dependence of the auxetic threshold on spacer length is attributed to the change in glass transition temperatures observed as the spacer length changes; specifically, the higher the *T*_g_, the higher the auxetic threshold. Evidence to support the dependence of the auxetic threshold on *T*_g_ comes from the convergence of auxetic thresholds (at strains around 0.56) upon analysis of the samples at the same reduced temperature relative to *T*_g_ (1.06 × *T*_g_(K)). However, the magnitude of the auxetic response, as measured by the lowest value of Poisson’s ratio observed during mechanical analysis, appears to be significantly smaller for the spacer containing three methylene units (Poisson’s ratio of −0.47) than it does for four, five and six methylene units (−0.95 to −1.20). This difference is attributed to a significant reduction in the length and flexibility of the side group with the spacer containing three methylene units, hindering the out-of-plane rotation of mesogens required for auxeticity. This suggests the magnitude of the response may also be tailored by changes in the chemical structure.

The results presented in this work are the first systematic attempts to determine structure–property relationships within auxetic LCEs. The information gleaned has given further understanding as to what aspects of the LCEs play a part in determining the magnitude of the auxetic response, as well as how the auxetic threshold may be adjusted. Such information will likely prove invaluable in facilitating the design of future generations of auxetic LCEs. However, there is still much further work to be undertaken to determine the effect of other changes in the molecular structure on auxetic LCEs, for example, the effect of changes to the mesogen structure or backbone chemistry.

## Figures and Tables

**Table 1:** The names assigned to the LCEs, based on the cyanobiphenyl monomer used in their synthesis.

LCE Name	Cyanobiphenyl Monomer	Spacer Length (Methylene Units)
AN03	A3OCB	3
AN04	A4OCB	4
AN05	A5OCB	5
AN06	A6OCB	6
AN07	A7OCB	7
AN08	A8OCB	8
AN09	A9OCB	9

**Table 2:** The effect of spacer length on the auxetic threshold of the LCEs at 22 °C; the glass transition temperatures (*T*_g_) of the LCEs; the temperatures at which mechanical measurements should be conducted to achieve the same reduced temperature relative to *T*_g_ as for AN06 (*T*_experiment_) and the auxetic thresholds observed for each LCE at the appropriate reduced temperature ^†^.

LCE	Spacer Length (Methylene Units)	Auxetic Threshold Strain (@ 22 °C)	*T*_g_ (°C)	*T*_experiment_ (°C)	Auxetic Threshold Strain (@ Reduced Temperature)
AN03	3	-	21	38	0.60 (±0.05)
AN04	4	0.81 (±0.05)	12	28	0.52 (±0.05)
AN05	5	0.65 (±0.05)	9	25	0.59 (±0.05)
AN06	6	0.56 (±0.05)	6	22	0.56 (±0.05)
AN07	7	-	2	18	-
AN08	8	-	−1	15	-
AN09	9	-	−2	14	-

^†^ ‘-’ indicates no auxetic response was observed prior to elastic failure.

## Data Availability

The data underlying this study are openly available in the dataset associated with “Structure-Property Relationships in Auxetic Liquid Crystal Elastomers—The Effect of Spacer Length”, available at https://doi.org/10.5518/1449.

## Supplementary Materials

The following supporting information can be downloaded at: https://www.mdpi.com/article/10.3390/polym16141957/s1: Additional experimental information, Figure S1: The clearing temperature of the LCE precursor mixtures displayed as a function of spacer length; Figure S2: The nematic liquid crystal textures observed for the LCE mixtures at room temperature (22 °C); Figure S3: One-dimensional SAXS and WAXS data for each of the LCE samples at 25 °C; Figure S4: Two-dimensional SAXS data obtained for the LCEs at 25 °C; Figure S5: Two-dimensional WAXS data obtained for the LCEs at 25 °C; Figures S6–S12: DSC thermograms for the LCEs; Figure S13: The effect of spacer length on the glass transition temperature of the LCEs; Figure S14: Thermal shape-change data for the LCEs; Figure S15: Order parameter measurements for the LCEs; Figures S16–S21: One- and two-dimensional X-ray scattering as a function of temperature for the LCEs. References [8,11,14,47,48] are cited in the supplementary materials.
