# Supplementary material for: Structure–Property Relationships in Auxetic Liquid Crystal Elastomers—The Effect of Spacer Length
Source: Polymers (Basel). 2024 Jul 9;16(14):1957. doi: 10.3390/polym16141957 (PMC11280505; doi:10.3390/polym16141957)
Supplement: Supplementary file 1 [file polymers-16-01957-s001.zip › polymers-3077560-supplementary.pdf]

Structure–Property Relationships in Auxetic Liquid Crystal Elastomers—The Effect of Spacer Length

Stuart R. Berrow [1,*], Thomas Raistrick [1], Richard J. Mandle [1,2] and Helen F. Gleeson [1]

[1] School of Physics and Astronomy, University of Leeds, Leeds LS2 9JT, UK
[2] School of Chemistry, University of Leeds, Leeds LS2 9JT, UK
* Correspondence: s.r.berrow@leeds.ac.uk

Electronic Supplementary Information

Supporting Information available: Experimental information, synthetic procedures for the synthesis of all monomers and precursors, phase transition behaviours for LCE precursor mixtures, one and two-dimensional X-ray scattering data for the LCEs, DSC thermograms for LCEs, thermally induced shape change measurements for the LCEs, order parameter measurements for the LCEs..

Table Of Contents

Experimental Information 2
Materials 2
Flash Chromatography 2
Monomer Synthesis 2
Elastomer Mold Fabrication (Homeotropic Alignment) 7
Liquid Crystal Elastomer Synthesis (Homeotropic Alignment) 7
Structural Analysis 8
Thermal Analysis 8
Optical Microscopy 9
X-Ray Scattering Measurements 9
Order Parameter Measurements 9
Mechanical Analysis 10
LCE Precursor Mixture Phase Analysis 11
One-Dimensional X-Ray Scattering 12
Two-Dimensional X-Ray Scattering 13
DSC Data - LCEs 15
Thermally Induced Shape Change Of LCEs 19
Order Parameter Measurements Of LCEs 20
Variable Temperature Small Angle X-Ray Scattering 21
References 26

Experimental Information

Materials

All materials were used as purchased without further purification, and were obtained from one of the following suppliers: Sigma Aldrich (Gillingham, UK), Fisher Scientific (Loughborough, UK), Apollo Scientific (Stockport, UK), Ambeed (Arlington Heights, IL, USA), Fluorochem (Glossop, UK), Tokyo Chemical Industry UK (Oxford, UK).

Flash Chromatography

Flash chromatography was performed on a Combiflash NextGen 300+ system (Teledyne Isco) using silica as a stationary phase (RediSep Silver), an appropriate mobile phase as specified in the experimental procedure, and detection in the 200-800 nm wavelength range.

Monomer Synthesis

Synthesis of Hydroxyl Terminated Alkoxy Cyanobiphenyls

The synthesis of 4′-(6-hydroxyhexyloxy)-[1,1′-biphenyl]-4-carbonitrile is given below as an example. A suspension of 4-cyano-4'-hydroxybiphenyl (6.61 g, 33.9 mmol), potassium carbonate (7.03 g, 50.9 mmol) and potassium iodide (0.23 g, 1.4 mmol) in DMF (50 mL) and was stirred at ambient temperature for 1 hour. To this suspension was added dropwise a solution of 6-chloro-1-hexanol (6.02 g, 44.1 mmol) in DMF (20 mL), and the resulting mixture stirred at 70 °C for 72 hours. The reaction mixture was then filtered under gravity, and the filtrate diluted in chloroform (2x 100 mL). The resulting solution was washed with water (100 mL), 1M sodium hydrogen carbonate solution (2x 100 mL) and saturated brine solution (100 mL) and the organic layer dried over sodium sulphate. The solvent was then removed under reduced pressure, and the resulting colourless solid recrystallised from 1:1 n-hexane:ethyl acetate yielding the product as colourless crystals (70% yield).

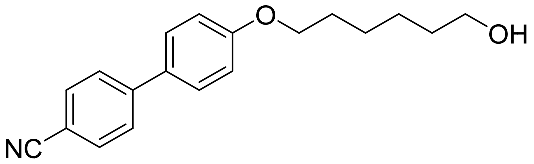

4′-(6-hydroxyhexyloxy)-[1,1′-biphenyl]-4-carbonitrile (6OCB-OH): Yield: 70% ^{1}H NMR (400 MHz, $CDCl_3$) δ_H(ppm): 1.39-1.51 (m, 4H, $-CH_2-$), 1.51-1.59 (m, 2H, $-CH_2-$), 1.72-1.81 (m, 2H, $-CH_2-$), 3.60 (t, 2H, *J*= 6.5 Hz, $-CH_2$-OH), 3.94 (t, 2H, *J*= 6.5 Hz, $-CH_2$-O-), 6.92 (ddd, 2H, *J*= 8.7, 3.0, 2.1 Hz, Ar-H), 7.45 (ddd, 2H, *J*= 8.7, 3.0, 2.1 Hz, Ar-H), 7.55-7.63 (m, 4H, Ar-H). ^{13}C{^{1}H} NMR (400 MHz, $CDCl_3$) δ_C(ppm): 25.6 ($-CH_2-$), 25.9 ($-CH_2-$), 29.2 ($-CH_2-$), 32.4 ($-CH_2-$), 62.3 (-O-CH_2-), 68.0 (-O-CH_2-), 110.1 (-C≡N), 115.1 (Ar C-H), 119.2 (Ar C-R), 127.2 (Ar C-H), 128.4 (Ar C-H), 131.4 (Ar C-R), 132.6 (Ar C-H), 145.3 (Ar C-R), 159.7 (Ar C-OR). m/z: 296.1643 ($M+H^+$).

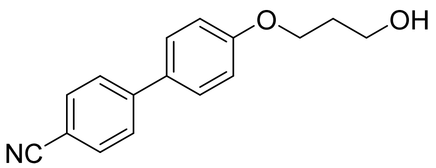

<u>4′-(3-hydroxypropyloxy)-[1,1′-biphenyl]-4-carbonitrile (3OCB-OH)</u>: Yield: 37%. ^{1}H NMR (400 MHz, $CDCl_3$) δ_H(ppm): 1.97-2.05 (m, 2H, -CH_2-), 3.80 (t, 2H, *J*= 6.0 Hz, -CH_2-OH), 4.11 (t, 2H, *J*= 6.0 Hz, -CH_2-O-), 6.94 (ddd, 2H, *J*= 8.9, 3.0, 2.1 Hz, Ar-H), 7.46 (ddd, 2H, *J*= 8.9, 3.0, 2.2 Hz, Ar-H), 7.54-7.64 (m, 4H, Ar-H). ^{13}C{^{1}H} NMR (400 MHz, $CDCl_3$) δ_C(ppm): 32.0 (-CH_2-), 60.4 (-O-CH_2-), 65.8 (-O-CH_2-), 110.1 (-C≡N), 115.1 (Ar C-H), 119.1 (Ar C-R), 127.1 (Ar C-H), 128.4 (Ar C-H), 131.7 (Ar C-R), 132.6 (Ar C-H), 145.2 (Ar C-R), 159.4 (Ar C-OR). m/z: 254.1173 (M+H$^+$).

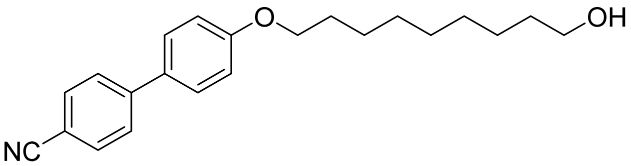

<u>4′-(9-hydroxynonyloxy)-[1,1′-biphenyl]-4-carbonitrile (9OCB-OH)</u>: Yield: 65% ^{1}H NMR (400 MHz, $CDCl_3$) δ_H(ppm): 1.22-1.55 (m, 12H, -CH_2-), 1.69-1.77 (m, 2H, -CH_2-), 3.57 (t, 2H, *J*= 6.8 Hz, -CH_2-OH), 3.94 (t, 2H, J= 6.8 Hz, -CH_2-O-), 6.92 (ddd, 2H, *J*= 8.8, 3.0, 2.0 Hz, Ar-H), 7.45 (ddd, 2H, *J*= 8.8, 2.0, 3.0 Hz, Ar-H), 7.54-7.64 (m, 4H, Ar-H). ^{13}C{^{1}H} NMR (400 MHz, $CDCl_3$) δ_C(ppm): 25.7 (-CH_2-), 26.0 (-CH_2-), 29.2 (-CH_2-), 29.3 (-CH_2-), 29.4 (-CH_2-), 29.5 (-CH_2-), 32.7 (-CH_2-), 63.1 (-O-CH_2-), 68.2 (-O-CH_2-), 110.1 (-C≡N), 115.1 (Ar C-H), 119.2 (Ar C-R), 127.1 (Ar C-H), 128.4 (Ar C-H), 131.3 (Ar C-R), 132.6 (Ar C-H), 145.3 (Ar C-R), 159.8 (Ar C-OR). m/z: 338.2113 (M+H$^+$).

Synthesis of Monomers from Hydroxyl Terminated Alkoxy Cyanobiphenyls

The synthesis of 6-(4-cyano-biphenyl-4′-yloxy)hexyl acrylate (A6OCB) is given below as an example. A solution of 4′-(6-hydroxyhexyloxy)-[1,1′-biphenyl]-4-carbonitrile (5.00 g, 16.9 mmol) and triethylamine (2.06 g, 20.3 mmol) in THF (50 mL) was cooled to 0 °C. To this solution was added dropwise a solution of acryloyl chloride (1.84 g, 20.3 mmol) in THF (10 mL). The resulting mixture as stirred at 0 °C for 1 hour, before stirring overnight at room temperature. The solvent was removed under reduced pressure, and the resulting solid dissolved in chloroform (100 mL), before washing with water (100 mL), 1M sodium hydrogen carbonate solution (100 mL) and saturated brine solution (100 mL). The organic layer was dried over sodium sulphate, and the solvent removed under reduced pressure. The resulting solid was recrystallised from 1:1 n-hexane:ethyl acetate to obtain product as a colourless solid (54% yield).

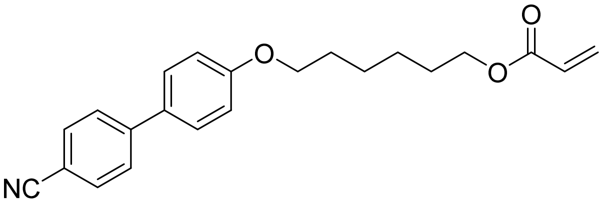

6-(4-Cyano-biphenyl-4′-yloxy)hexyl acrylate (A6OCB): Yield: 54%. ^{1}H NMR (400 MHz, $CDCl_3$) δ_H(ppm): 1.35-1.52 (m, 4H, -CH_2-), 1.61-1.70 (m, 2H, -CH_2-), 1.72-1.90 (m, 2H, -CH_2-), 3.94 (t, 2H, *J*= 6.5 Hz, -CH_2-O-), 4.10 (t, 2H, *J*= 6.5 Hz, -CH_2-O-), 5.74 (dd, 1H, *J*= 10.5, 0.8 Hz, =CH_2), 6.05 (dd, 1H *J*= 17.4 ,10.5 Hz, -CH=), 6.33 (dd, 1H, *J*= 10.5,0.8 Hz, =CH_2), 6.92 (ddd 2H, *J*= 8.8, 2.8, 2.1 Hz, Ar-H), 7.45 (ddd, 2H, *J*= 8.8, 2.8, 2.1 Hz, Ar-H), 7.54-7.64 (m, 4H, Ar-H). ^{13}C{^{1}H} NMR (400 MHz, $CDCl_3$) δ_C(ppm): 25.7 (-CH_2-), 28.6 (-CH_2-), 29.1 (-CH_2-), 64.5 (-O-CH_2-), 68.0 (-CH_2-O), 110.1 (-C≡N), 115.1 (Ar C-H), 119.2 (Ar C-R), 127.1 (Ar C-H), 128.3 (Ar C-H), 128.6 (C=C), 130.6 (C=C), 131.4 (Ar C-R), 132.6 (Ar C-H), 145.3 (Ar C-R), 159.8 (Ar C-OR), 166.4 (C=O). m/z: 350.1751 (M+H$^+$), 372.1573 (M+Na$^+$).

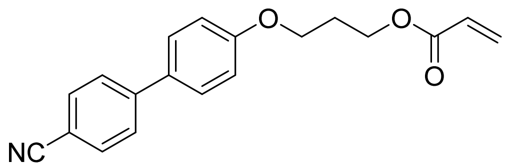

3-(4-Cyano-biphenyl-4′-yloxy)propyl acrylate (A3OCB): Yield: 50% ^{1}H NMR (400 MHz, $CDCl_3$) δ_H(ppm): 2.13 (quint, 2H, *J*= 6.2 Hz, -CH_2-), 4.05 (t, 2H, *J*= 6.1 Hz, -CH_2-O-), 4.31 (t, 2H, *J*= 6.3 Hz, -CH_2-O-), 5.77 (dd, 1H, *J*= 10.5,1.5 Hz, =CH_2), 6.06 (dd, 1H *J*= 17.3,10.5 Hz, -CH=), 6.35 (dd, 1H, *J*= 17.3,1.5 Hz, =CH_2), 6.92 (ddd, 2H, *J*= 8.8, 3.0, 2.1 Hz, Ar-H), 7.45 (ddd, 2H, *J*= 8.8, 3.0, 2.1 Hz, Ar-H), 7.54-7.64 (m, 4H, Ar-H). ^{13}C{^{1}H} NMR (400 MHz, $CDCl_3$) δ_C(ppm): 28.9 (-CH_2), 61.2 (-O-CH_2-), 64.5 (-CH_2-O), 110.2 (-C≡N), 115.1 (Ar C-H), 119.1 (Ar C-R) 127.1 (Ar C-H), 128.3 (Ar C-H), 128.4 (C=C), 130.9 (C=C), 131.7 (Ar C-R), 132.6 (Ar C-H), 145.2 (Ar C-R), 159.4 (Ar C-OR), 166.2 (C=O). m/z: 308.1281 (M+H$^+$), 330.1104 (M+Na$^+$).

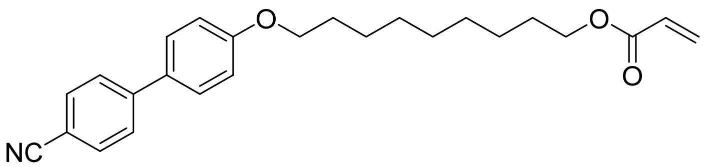

9-(4-Cyano-biphenyl-4′-yloxy)nonyl acrylate (A9OCB): Yield: 25% ^{1}H NMR (400 MHz, $CDCl_3$) δ_H(ppm): 1.24-1.45 (m, 10H, -CH_2-), 1.56-1.64 (m, 2H, -CH_2-), 1.70-1.77 (m, 2H, -CH_2-), 3.93 (t, 2H, *J*= 6.5 Hz, -CH_2-O-), 4.09 (t, 2H, *J*= 6.7 Hz, -CH_2-O-), 5.74 (dd, 1H, *J*= 10.4,1.5 Hz, =CH_2), 6.05 (dd, 1H *J*= 17.3,10.4 Hz, -CH=), 6.33 (dd, 1H, *J*= 17.3,1.5 Hz, =CH_2), 6.92 (ddd, 2H, *J*= 8.9, 3.0, 2.1 Hz, Ar-H), 7.45 (ddd, 2H, *J*= 8.9, 3.0, 2.1 Hz, Ar-H), 7.54-7.64 (m, 4H, Ar-H). ^{13}C{^{1}H} NMR (400 MHz, $CDCl_3$) δ_C(ppm): 25.9 (-CH_2), 26.0 (-CH_2), 28.6 (-CH_2), 29.2 (-CH_2-), 29.2 (-CH_2), 29.3 (-CH_2), 29.4 (-CH_2), 64.7 (-O-CH_2-), 68.2 (-CH_2-O), 110.2 (-C≡N), 115.1 (Ar C-H), 119.1 (Ar C-R), 127.1 (Ar C-H), 128.3 (Ar C-H), 128.7 (C=C), 130.5 (C=C), 131.3 (Ar C-R), 132.6 (Ar C-H), 145.3 (Ar C-R), 159.8 (Ar C-OR), 166.4 (C=O). m/z: 392.2226 (M+H$^+$), 414.2043 (M+Na$^+$).

Synthesis of Hydroxyl Terminated Alkyl Acrylates

The synthesis of 8-hydroxyoctyl acrylate is given below as a representative example. To dichloromethane (100 mL) was added 1,8-octanediol (7.30 g, 50.0 mmol), 1-(3-Dimethylaminopropyl)-3-ethylcarbodiimide hydrochloride (4.79 g, 25.0 mmol) and 4-(Dimethylamino)pyridine (0.61 g, 5.0 mmol). To this solution was added dropwise acrylic

acid (1.80 g, 25.0 mmol), and the solution stirred at room temperature overnight. The mixture was concentrated under reduced pressure, and purified over silica gel, using a gradient elution of 8:2 hexane:ethyl acetate to 6:4 hexane:ethyl acetate. The product was isolated as a colourless liquid (40% yield).

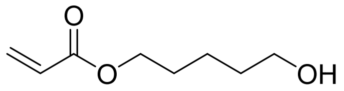

5-hydroxpentyl acrylate: Yield: 30%. ^{1}H NMR (400 MHz, $CDCl_3$) δ_H(ppm): 1.37-1.44 (m, 4H, -CH_2-), 1.51-1.60 (m, 2H, -CH_2-), 1.61-1.69 (m, 2H, -CH_2-), 3.60 (t, 2H, *J*= 6.5 Hz, -CH_2-O-), 4.10 (t, 2H, *J*= 6.6 Hz, -CH_2-O-), 5.77 (dd, 1H, *J*= 10.4,1.5 Hz, =CH_2), 6.06 (dd, 1H *J*= 17.3,10.4 Hz, -CH=), 6.35 (dd, 1H, *J*= 17.4,1.5 Hz, =CH_2). $^{13}C\{^{1}H\}$ NMR (400 MHz, $CDCl_3$) δ_C(ppm): 22.4 (-CH_2-), 28.5 (-CH_2-), 32.3 (-CH_2-), 62.7 (-CH_2-O), 64.5 (-CH_2-O-), 128.6 (C=C), 130.6 (C=C), 166.4 (C=O). m/z: 181.0833 ($M+Na^+$).

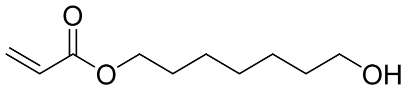

7-hydroxyheptyl acrylate: Yield: 44%. ^{1}H NMR (400 MHz, $CDCl_3$) δ_H(ppm): ^{1}H NMR (400 MHz, $CDCl_3$) δ_H(ppm): 1.27-1.38 (m, 8H, -CH_2-), 1.47-1.54 (m, 2H, -CH_2-), 1.57-1.65 (m, 2H, -CH_2-), 3.57 (t, 2H, *J*= 6.6 Hz, -CH_2-O-), 4.08 (t, 2H, *J*= 6.7 Hz, -CH_2-O-), 5.75 (dd, 1H, *J*= 10.4,1.5 Hz, =CH_2), 6.05 (dd, 1H *J*= 17.3,10.4 Hz, -CH=), 6.33 (dd, 1H, *J*= 17.3,1.5 Hz, =CH_2). $^{13}C\{^{1}H\}$ NMR (400 MHz, $CDCl_3$) δ_C(ppm): 25.6 (-CH_2-), 25.9 (-CH_2-), 28.5 (-CH_2-), 29.0 (-CH_2-), 32.7 (-CH_2-), 63.0 (-CH_2-O), 64.6 (-CH_2-O-), 128.6 (C=C), 130.4 (C=C), 166.4 (C=O). m/z: 187.1328 ($M+H^+$), 209.1147 ($M+Na^+$).

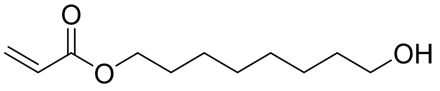

8-hydroxyoctyl acrylate: Yield: 40%. ^{1}H NMR (400 MHz, $CDCl_3$) δ_H(ppm): ^{1}H NMR (400 MHz, $CDCl_3$) δ_H(ppm): 1.23-1.35 (m, 8H, -CH_2-), 1.46-1.53 (m, 2H, -CH_2-), 1.56-1.64 (m, 2H, -CH_2-), 3.57 (t, 2H, *J*= 6.6 Hz, -CH_2-O-), 4.08 (t, 2H, *J*= 6.9 Hz, -CH_2-O-), 5.75 (dd, 1H, *J*= 10.4,1.5 Hz, =CH_2), 6.05 (dd, 1H *J*= 17.3,10.4 Hz, -CH=), 6.32 (dd, 1H, *J*= 17.4,1.5 Hz, =CH_2). $^{13}C\{^{1}H\}$ NMR (400 MHz, $CDCl_3$) δ_C(ppm): 25.7 (-CH_2-), 25.9 (-CH_2-), 28.6 (-CH_2-), 29.2 (-CH_2-), 29.3 (-CH_2-), 32.8 (-CH_2-), 63.0 (-CH_2-O), 64.7 (-CH_2-O-), 128.6 (C=C), 130.5 (C=C), 166.4 (C=O). m/z: 201.1483 ($M+H^+$), 223.1303 ($M+Na^+$).

Synthesis of Monomers from Hydroxyl Terminated Alkyl Acrylates

The synthesis of 4-(4-Cyano-biphenyl-4′-yloxy)butyl acrylate (A4OCB) is given below as a representative example. To anhydrous tetrahydrofuran (50 mL) was added 4-hydroxy-4′-cyanobiphenyl (3.60 g, 18.4 mmol), triphenylphosphine (6.77 g, 25.8 mmol) and 4-hydroxybutyl acrylate (3.72 g, 25.8 mmol). The solution was degassed with nitrogen and cooled in an ice bath, before diisopropyl azodicarboxylate (5.22 g, 25.8 mmol) was added dropwise, and the resulting solution stirred at room temperature overnight. The solvent was removed under reduced pressure, and the resulting mixture purified over silica gel, using

8:2 hexane:ethyl acetate as eluent. The resulting material was then recrystallised from isopropanol to yield the product as colourless crystals (62% yield).

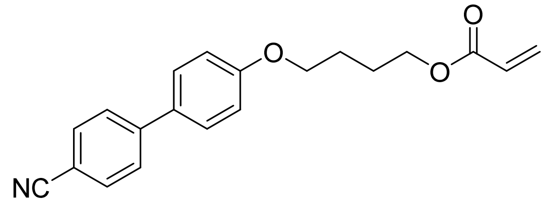

<u>4-(4-Cyano-biphenyl-4′-yloxy)butyl acrylate (A4OCB)</u>: Yield: 62% ^{1}H NMR (400 MHz, CDCl$_3$) δ$_H$(ppm): 1.80-1.87 (m, 4H, -CH$_2$-), 3.98 (t, 2H, *J*= 5.8 Hz, -CH$_2$-O-), 4.19 (t, 2H, *J*= 5.8 Hz, -CH$_2$-O-), 5.76 (dd, 1H, *J*= 10.4,1.5 Hz, =CH$_2$), 6.06 (dd, 1H *J*= 17.3,10.5 Hz, -CH=), 6.35 (dd, 1H, *J*= 17.3,1.5 Hz, =CH$_2$), 6.92 (ddd, 2H, *J*= 8.8, 3.0, 2.1 Hz, Ar-H), 7.46 (ddd, 2H, *J*= 8.8, 3.0, 2.0 Hz, Ar-H), 7.54-7.64 (m, 4H, Ar-H). ^{13}C{^{1}H} NMR (400 MHz, CDCl$_3$) δ$_C$(ppm): 25.5 (-CH$_2$), 25.9 (-CH$_2$), 64.1 (-O-CH$_2$-), 67.4 (-CH$_2$-O), 110.2 (-C≡N), 115.1 (Ar C-H), 119.1 (Ar C-R), 127.1 (Ar C-H), 128.3 (Ar C-H), 128.5 (C=C), 130.8 (C=C), 131.6 (Ar C-R), 132.6 (Ar C-H), 145.3 (Ar C-R), 159.6 (Ar C-OR), 166.3 (C=O). m/z: 322.1439 (M+H$^+$), 344.1257 (M+Na$^+$).

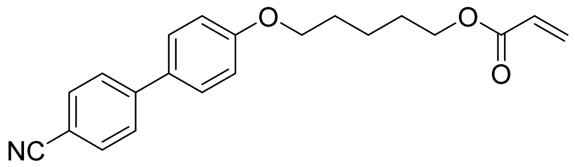

<u>5-(4-Cyano-biphenyl-4′-yloxy)pentyl acrylate (A5OCB)</u>: Yield: 63%. ^{1}H NMR (400 MHz, CDCl$_3$) δ$_H$(ppm): 1.47-1.56 (m, 2H, -CH$_2$-), 1.66-1.74 (m, 2H, -CH$_2$-), 1.75-1.83 (m, 2H, -CH$_2$-), 3.95 (t, 2H, *J*= 6.3 Hz, -CH$_2$-O-), 4.13 (t, 2H, *J*= 6.6 Hz, -CH$_2$-O-), 5.75 (dd, 1H, *J*= 10.4,1.5 Hz, =CH$_2$), 6.05 (dd, 1H *J*= 17.3,10.4 Hz, -CH=), 6.34 (dd, 1H, *J*= 17.3,1.5 Hz, =CH$_2$), 6.91 (ddd, 2H, *J*= 8.9,3.0,2.1 Hz, Ar-H), 7.45 (ddd, 2H, *J*= 8.8,3.0,2.0 Hz, Ar-H), 7.54-7.64 (m, 4H, Ar-H). ^{13}C{^{1}H} NMR (400 MHz, CDCl$_3$) δ$_C$(ppm): 22.7 (-CH$_2$), 28.4 (-CH$_2$-), 28.9 (-CH$_2$), 64.4 (-O-CH$_2$-), 67.8 (-CH$_2$-O), 110.2 (-C≡N), 115.1 (Ar C-H), 119.1 (Ar C-R), 127.2 (Ar C-H), 128.5 (Ar C-H), 128.6 (C=C), 130.7 (C=C), 131.4 (Ar C-R), 132.6 (Ar C-H), 145.3 (Ar C-R), 159.7 (Ar C-OR), 166.4 (C=O). m/z: 336.1593 (M+H$^+$), 358.1417 (M+Na$^+$).

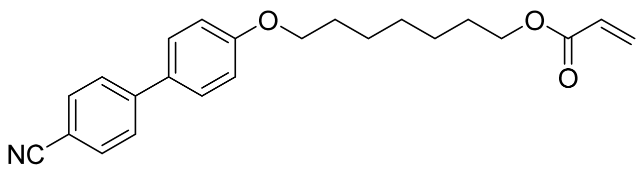

<u>7-(4-Cyano-biphenyl-4′-yloxy)heptyl acrylate (A7OCB)</u>: Yield: 86%. %. ^{1}H NMR (400 MHz, CDCl$_3$) δ$_H$(ppm): 1.33-1.47 (m, 6H, -CH$_2$-), 1.58-1.66 (m, 2H, -CH$_2$-), 1.70-1.78 (m, 2H, -CH$_2$-), 3.93 (t, 2H, *J*= 6.5 Hz, -CH$_2$-O-), 4.10 (t, 2H, *J*= 6.7 Hz, -CH$_2$-O-), 5.75 (dd, 1H, *J*= 10.4,1.5 Hz, =CH$_2$), 6.05 (dd, 1H *J*= 17.3,10.4 Hz, -CH=), 6.33 (dd, 1H, *J*= 17.3,1.5 Hz, =CH$_2$), 6.92 (ddd, 2H, *J*= 8.8,3.0,2.1 Hz, Ar-H), 7.46 (ddd, 2H, *J*= 8.8,3.0,2.1 Hz, Ar-H), 7.54-7.64 (m, 4H, Ar-H). ^{13}C{^{1}H} NMR (400 MHz, CDCl$_3$) δ$_C$(ppm): 25.9 (-CH$_2$), 26.0 (-CH$_2$), 28.6 (-CH$_2$-), 29.0 (-CH$_2$), 29.2 (-CH$_2$), 64.6 (-O-CH$_2$-), 68.1 (-CH$_2$-O), 110.1 (-C≡N), 115.1 (Ar C-H), 119.1 (Ar C-R), 127.2

(Ar C-H), 128.4 (Ar C-H), 128.7 (C=C), 130.5 (C=C), 131.4 (Ar C-R), 132.6 (Ar C-H), 145.3 (Ar C-R), 159.8 (Ar C-OR), 166.4 (C=O). m/z: 364.1907 ($M+H^+$), 386.1727 ($M+Na^+$).

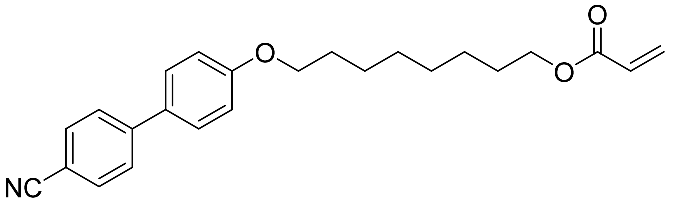

8-(4-Cyano-biphenyl-4′-yloxy)octyl acrylate (A8OCB): Yield: 62%. ^{1}H NMR (400 MHz, $CDCl_3$) δ_H(ppm): 1.27-1.45 (m, 8H, $-CH_2-$), 1.57-1.65 (m, 2H, $-CH_2-$), 1.71-1.79 (m, 2H, $-CH_2-$), 3.93 (t, 2H, *J*= 6.5 Hz, $-CH_2-O-$), 4.09 (t, 2H, *J*= 6.7 Hz, $-CH_2-O-$), 5.74 (dd, 1H, *J*= 10.4,1.5 Hz, $=CH_2$), 6.05 (dd, 1H *J*= 17.3,10.4 Hz, -CH=), 6.33 (dd, 1H, *J*= 17.3,1.5 Hz, $=CH_2$), 6.92 (ddd, 2H, *J*= 8.8, 3.0, 2.1 Hz, Ar-H), 7.45 (ddd, 2H, *J*= 8.8, 3.0, 2.1 Hz, Ar-H), 7.54-7.64 (m, 4H, Ar-H). $^{13}C\{^1H\}$ NMR (400 MHz, $CDCl_3$) δ_C(ppm): 25.9 ($-CH_2$), 26.0 ($-CH_2$), 28.6 ($-CH_2-$), 28.9 ($-CH_2$), 29.1-29.2 ($-CH_2$), 64.6 ($-O-CH_2-$), 68.1 ($-CH_2-O$), 110.1 (-C≡N), 115.2 (Ar C-H), 119.1 (Ar C-R), 127.1 (Ar C-H), 128.3 (Ar C-H), 128.6 (C=C), 130.5 (C=C), 131.3 (Ar C-R), 132.6 (Ar C-H), 145.3 (Ar C-R), 159.8 (Ar C-OR), 166.4 (C=O). m/z: 378.2067 ($M+H^+$), 400.1887 ($M+Na^+$).

Elastomer Mold Fabrication (Homeotropic Alignment)

The LCE’s were synthesized in bespoke alignment molds, which were made in accordance with previous literature.[1] A glass slide coated in indium-tin oxide (ITO) (5 cm x 2.5 cm), and an ITO-coated poly(ethylene terephthalate) (PET) substrate (5 cm x 2.5 cm) (DuPont Teijin Films, Redcar, UK) were spin coated on the ITO surface with an aqueous 0.5 wt % cetyl trimethyl ammonium bromide (CTAB) solution. These two substrates were then adhered, via Melinex® 401 spacers (5 cm x 0.2 cm x 100 μm) (DuPont Teijin Films, Redcar, UK) and UVS-91 adhesive, so that the CTAB coated surfaces were the inner surfaces of the constructed cell. The adhesive was then cured by irradiation under 350 nm (2.5 Wcm-2) for 10 minutes, to yield the constructed LCE mold with a gap of ~100 μm thickness.

Liquid Crystal Elastomer Synthesis (Homeotropic Alignment)

In a typical procedure, RM82 (3.5 mol%), the cyanobiphenyl monomer AnOCB (24.4 mol%), and 6OCB (54.6 mol%) were heated to 120 °C with stirring until a homogeneous isotropic phase was obtained. The mixture was cooled to 50 °C, followed by the addition of EHA (16 mol%) and MBF (1.5 mol%), and stirred for 5 minutes. The mixture was then filled into a mold at 50 °C via pipette, before being cooled to room temperature. A voltage of 50 V_{rms} at 1 kHz was applied to cell to enhance the homeotropic alignment, and the samples were then cured under 350 nm (2.5 Wcm^{-2}) irradiation for 2 hours. After curing, the samples were removed from the molds (using a small amount of isopropanol if necessary to aid delamination from the substrates), and left to stand in a solution of dichloromethane(DCM):isopropanol (30:70) overnight to remove the non-reactive 6OCB. The samples were then allowed to dry under ambient conditions for 5 hours, to yield the final LCE films.

Structural Analysis

Nuclear magnetic resonance (NMR) spectra were recorded using a Bruker AVANCE III (400 MHz) NMR spectrometer (Bruker UK Ltd., Coventry, UK) at 298 K and referenced to TMS. NMR spectra were viewed and analysed using MNova NMR software.

Accurate Mass spectra were acquired on a Bruker Impact II QqTOF spectrometer equipped with a VIPHESI source using electrospray ionisation. Samples were introduced using an HTC PAL autosampler and Bruker Elute Pump. HPLC columns were heated to 40 °C unless otherwise stated. Samples passed through a Bruker Diode array UV-detector before entering the mass spectrometer. Calibration was performed by infusion of 5mM sodium formate solution at the end of each acquisition. Samples were submitted as solutions in acetonitrile at 10 µg/mL concentration, and data were collected in positive mode.

Thermal Analysis

Differential scanning calorimetry (DSC) measurements were performed using a TA Instruments Q2000 DSC instrument (TA Instruments, Wilmslow UK), equipped with a RCS90 Refrigerated cooling system (TA Instruments, Wilmslow UK). The instrument was calibrated against an Indium standard, and data were processed using TA Instruments Universal Analysis Software. Samples were analysed under a nitrogen atmosphere, in hermetically sealed aluminium TZero crucibles (TA Instruments, Wilmslow, UK) and subjected to three analysis cycles.

For the analysis of the synthesised monomers (and intermediates) each cycle consisted of: a heating phase from 0–140 °C at a heating rate of 10 °C/min, an isothermal phase at 140 °C for 2 minutes, a cooling phase from 140–0 °C at 10 °C/min, and an isothermal phase at 0 °C for 2 minutes. Melting points are reported as peak values on the first heating cycle, and clearing temperatures are reported as onset temperatures on the first cooling cycle.

For the analysis of the uncured LCE mixtures, each cycle consisted of: a heating phase from 0–100 °C at a heating rate of 10 °C/min, an isothermal phase at 100 °C for 2 minutes, a cooling phase from 100–0 °C at 10 °C/min, and an isothermal phase at 0 °C for 2 minutes. Clearing temperatures are reported as onset values.

For LCE analysis, each cycle consisted of: a heating phase from -50–150 °C at a heating rate of 10 °C/min, and isothermal phase at 150 °C for 2 minutes, a cooling phase from 150 – -50 °C at 10 °C/min, and an isothermal phase at -50 °C for 2 minutes. The glass transition temperatures of the polymers were recorded as the onset value, on the heating phase of the second cycle.

The thermally induced shape change of the LCEs was monitored using a bespoke set up. The sample was mounted onto a glass cover slip, with a small amount of silicon oil used to aid thermal contact and minimise the opportunity for friction to prevent shape change. The cover slip was then mounted onto a hot stage. The sample was heated from 25 °C to 200 °C at a heating rate of 1 °C/min, and an image of the sample was recorded every 5 minutes.

The sample length parallel to the director was then recorded using imageJ software, with an average value taken from three points across the sample at each temperature.

Optical Microscopy

Polarised light optical microscopy (POM) was performed using a Leica DM2700P polarised light microscope (Leica Microsystems (UK) Ltd., Milton Keynes, UK), equipped with 10x and 50x magnification, and a rotatable stage. For LCE samples, the films were mounted on a glass slide, and analysed under ambient conditions, using 10x magnification. For phase identification of the liquid crystalline materials and mixtures, a Mettler Toledo FP82HT Hot Stage (Mettler-Toledo Ltd., Leicester, UK), controlled by a Mettler Toledo FP90 central processor (Mettler-Toledo Ltd., Leicester, UK) was used to control the temperature of the sample with a relative accuracy of 0.1 °C. In this case, the sample was mounted between a glass microscope slide and a glass cover slip, and samples were analysed using 50x magnification. Images were recorded using a Nikon D3500 Digital Camera (Nikon UK Ltd., Surbiton, UK), using DigiCamControl software.

Conoscopy was performed using a Leica DM 2700P polarizing microscope in transmission mode under cross-polarized conditions. The microscope was equipped with a 0.9 numerical aperture (NA) condensing lens and a 0.95 NA 80x Leitz microscope objective. To study the conoscopic patterns, a Bertrand lens was inserted between the microscope objective and the eyepiece. The conoscopic patterns were captured with a Nikon D3500 camera. The homeotropic sample is held under strain with Kapton tape and allowed to stress relax for two minutes before the image of the conoscopic pattern is recorded. The strain is applied parallel to the analyzer of the cross-polarized microscope and measured with digital calipers.

X-ray Scattering Measurements

2D Small angle (SAXS) and wide angle (WAXS) X-ray scattering experiments were performed on an Anton Paar SAXSpoint 5.0 system (K-α Cu source, λ=1.5418 Å) with a Dectris EIGER2 R 1M (1028 pixel x 1062 pixel array). Measurements were performed at room temperature on 100 μm (nominal thickness) LCE samples averaging 5 frames with exposures times of 60 s. A 2 mm beam size was used and the measurements were run using a beam stop-less set-up. A background scan was performed for both the SAXS and WAXS detector position which was subtracted from the measurements to minimise contributions from intrinsic background scattering and the Mylar protective film in front of the detector. 2D data reduction was performed by radially integrating the 2D patterns whilst masking the central contribution related to the non-scattered X-ray beam.

Order Parameter Measurements

The nematic order parameter of the liquid crystal elastomers was recorded using Raman spectroscopy as described previously.[1,2] Raman spectra were recorded using a Renishaw inVia Raman spectrometer, equipped with a 532 nm, 50 mW solid state laser, and a Leica DM2700P polarised light microscope equipped with a rotating stage. Measurements were

made using a 20x objective with 1% laser power, and the sample rotated in 10° steps. In brief, the full depolarisation ratio was recorded, allowing the order parameters to be deduced from fits to the depolarisation ratio data. The 1606 cm^{-1} Raman mode, which is associated with the C-C stretch of the biphenyl rings of the mesogenic units, was selected for analysis. The order parameter was deduced with an accuracy of ±0.05

Mechanical Analysis

Mechanical measurements were conducted using bespoke equipment designed and manufactured in-house, full specifications for which can be found in previous work. [2,3] This apparatus consists of two actuators and a load cell, enclosed within a temperature-controlled environment, and is equipped with optics that enable images of the sample to be recorded both via optical microscopy and polarising optical microscopy simultaneously. In this work, samples of 20 mm x 2 mm were analysed at room temperature. The initial gap between the actuators was 16.5 mm, and the samples were subject to strain steps of 0.5 mm at 10-minute intervals, until the sample failed. The samples were strained perpendicular to the initial nematic director, as displayed in Figure 5 in the article text. Particle tracking was used to calculate strains in all three dimensions, assuming a 2-pixel uncertainty at each point, in accordance with previously reported procedures.[3]

Density Functional Theory Calculations

Electronic structure calculations were performed using the Gaussian G16 revision C01 software package. We employed the B3LYP hybrid DFT functional and the cc-pVTZ basis set.[4,5] Following geometry optimisation, we performed a frequency calculation to confirm the attained geometry to be at a minimum on the potential energy surface. The maximum end-to-end distance of each molecule was computed from the atomic coordinates.

LCE Precursor Mixture Phase Analysis

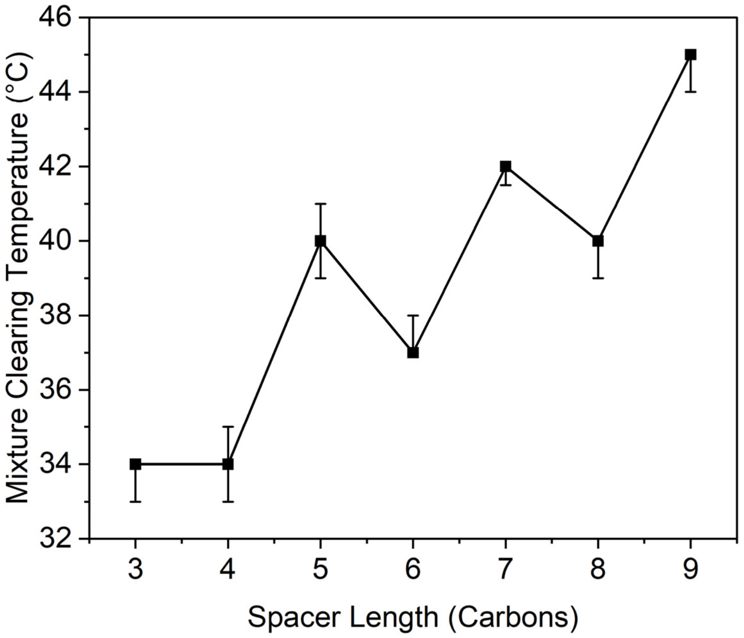

Figure S1. The clearing temperature of the LCE precursor mixtures displayed as a function of spacer *length*.

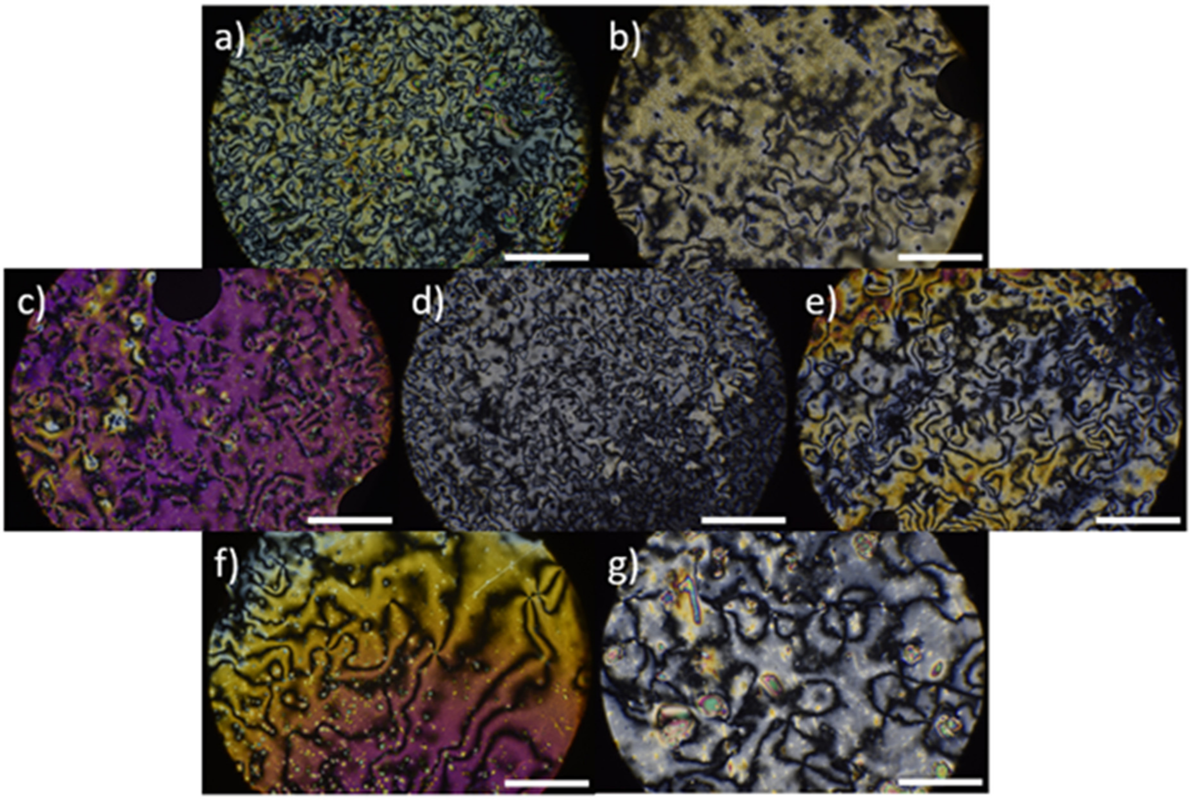

Figure S2. The nematic liquid crystal textures observed for the LCE mixtures at room temperature (22 °C). In all cases the scale bar represents 100 μm. The LCE mixture displayed in each image is: a) AN03, b) AN04, c) AN05, d) AN06, e) AN07, f) AN08 and g) AN09. The

samples are held between cover slips with no surface alignment as this allows clearer visualisation of the schlieren textures than is possible in a highly aligned sample.

One-Dimensional X-Ray Scattering

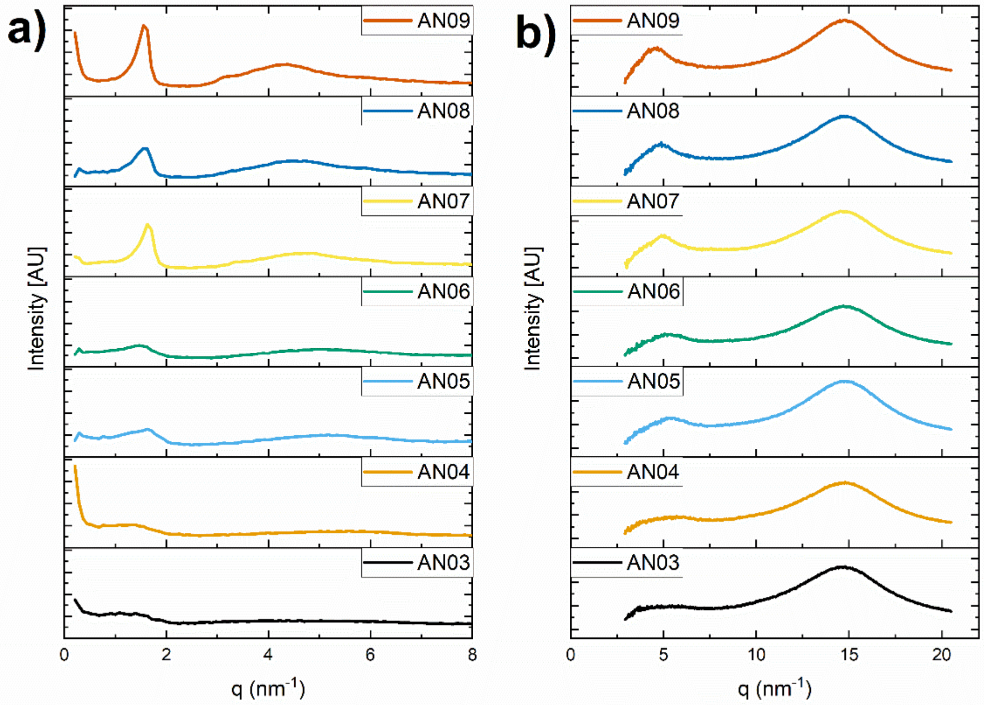

Figure S3. One dimensional a) SAXS and b) WAXS data for each of the LCE samples at 25 °C. All samples were approximately 100 μm, and were analysed at 25 °C. The intensity scales for all samples in graphs a) and b) are comparable [a) 0-0.7 AU and b) 0-3.825 AU].

Two-Dimensional X-Ray Scattering

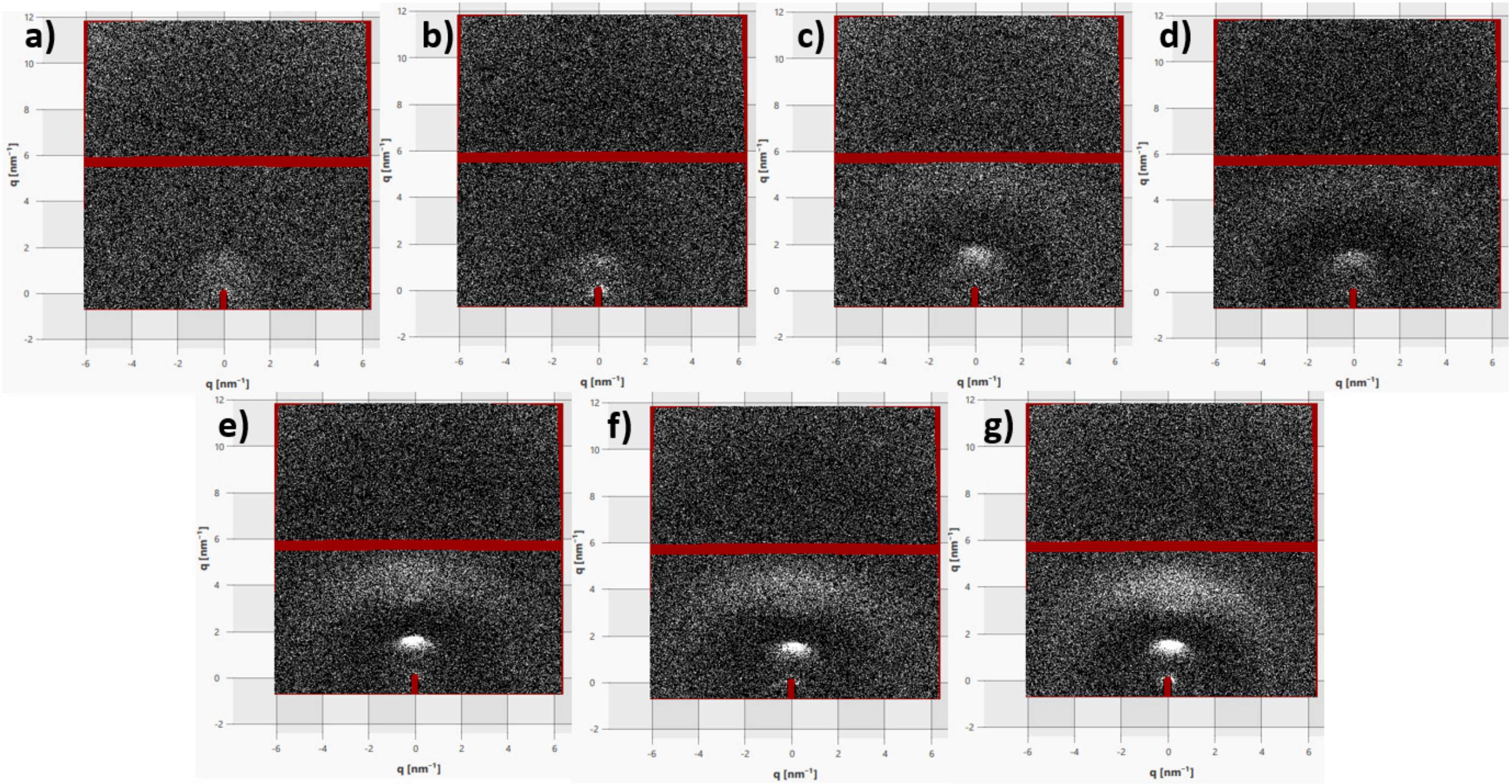

Figure S4. Two-dimensional SAXS data obtained for a) AN03, b) AN04, c) AN05, d) AN06, e) AN07, f) AN08 and g) AN09 LCEs at 25 °C.

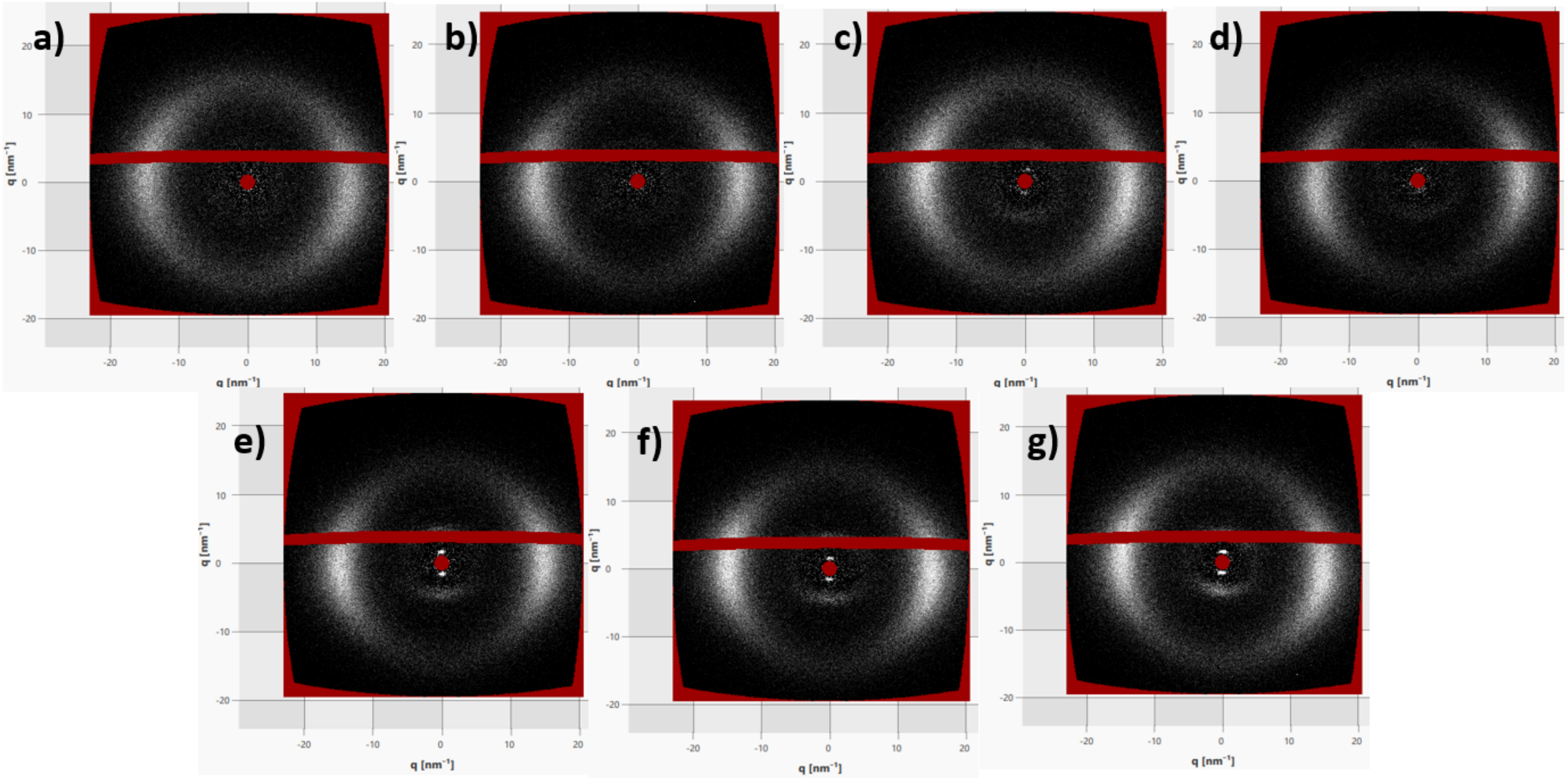

Figure S5 - Two-dimensional WAXS data obtained for a) AN03, b) AN04, c) AN05, d) AN06, e) AN07, f) AN08 and g) AN09 LCEs at 25 °C.

DSC Data - LCEs

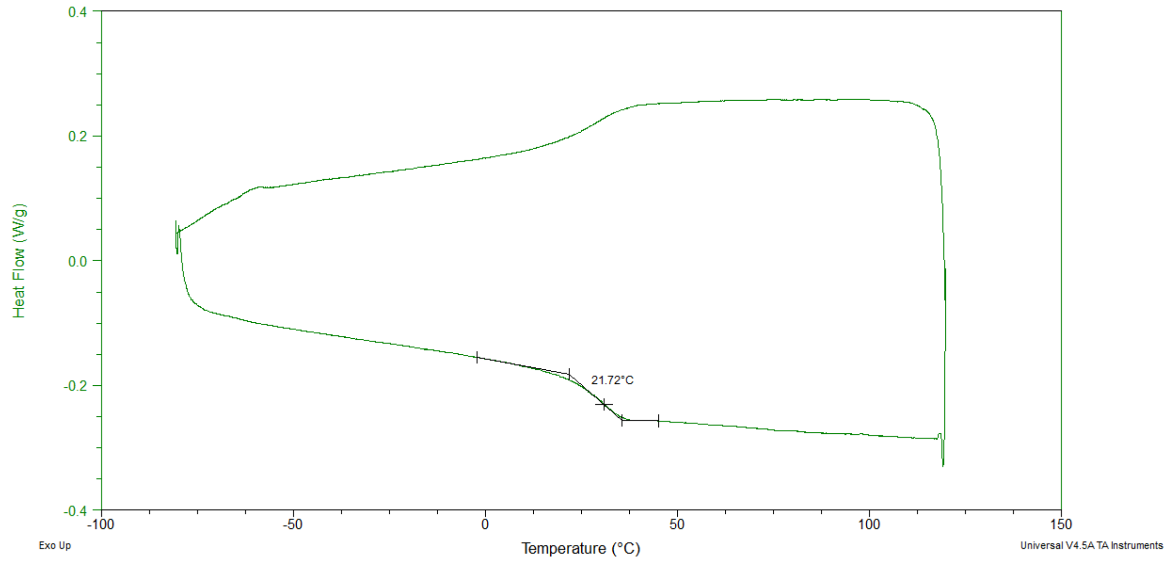

Figure S6. An example DSC Thermogram for the AN03 elastomer, showing the second heat/cool cycle, with exothermic transitions orientated upwards.

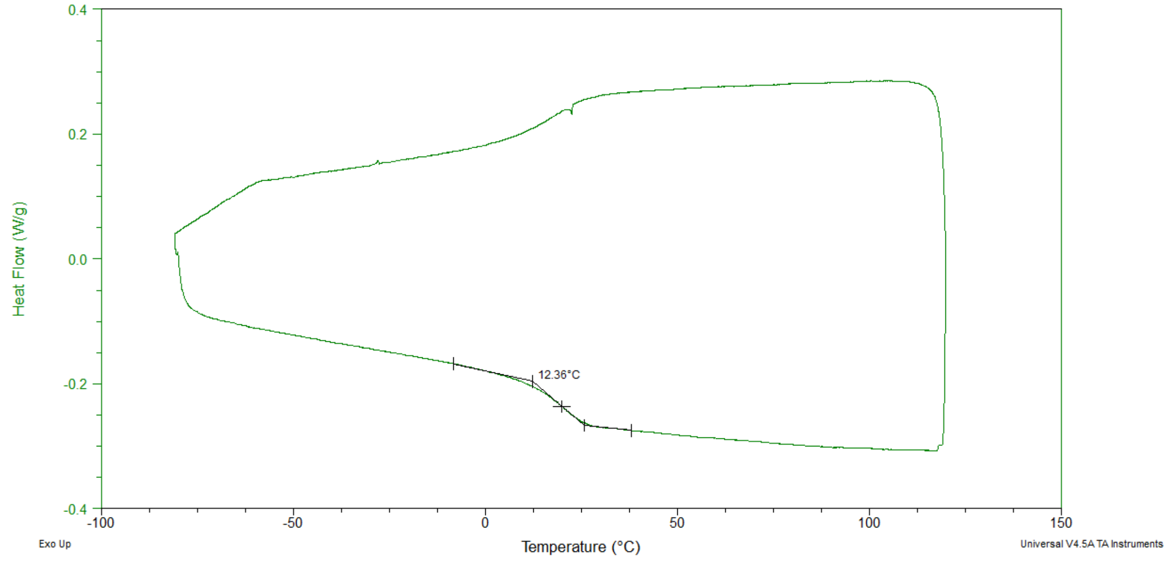

Figure S7. An example DSC Thermogram for the AN04 elastomer, showing the second heat/cool cycle, with exothermic transitions orientated upwards.

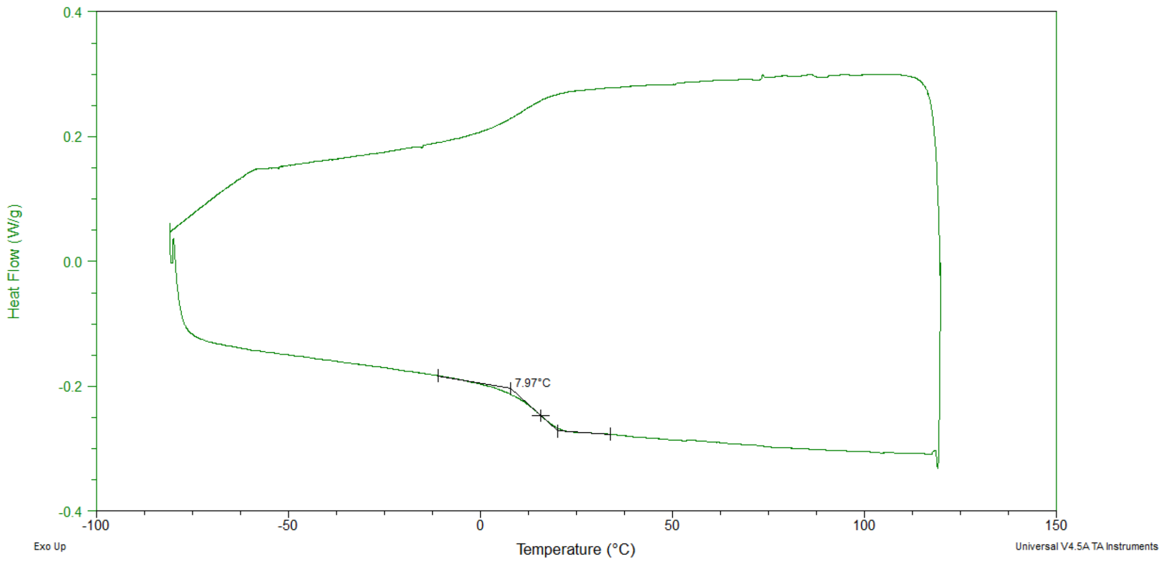

Figure S8. An example DSC Thermogram for the AN05 elastomer, showing the second heat/cool cycle, with exothermic transitions orientated upwards.

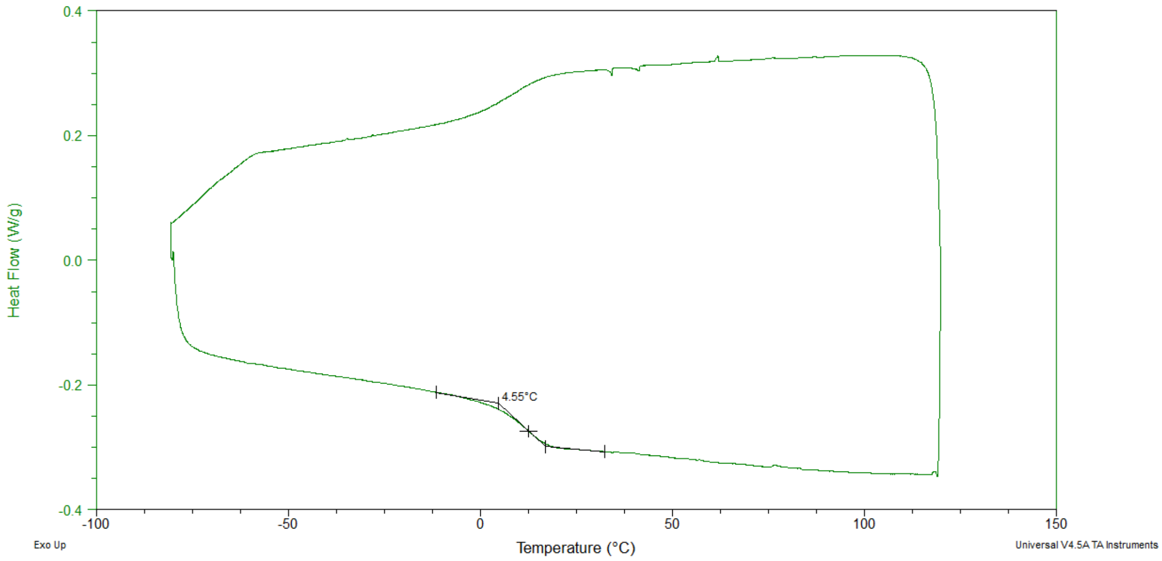

Figure S9. An example DSC Thermogram for the AN06 elastomer, showing the second heat/cool cycle, with exothermic transitions orientated upwards.

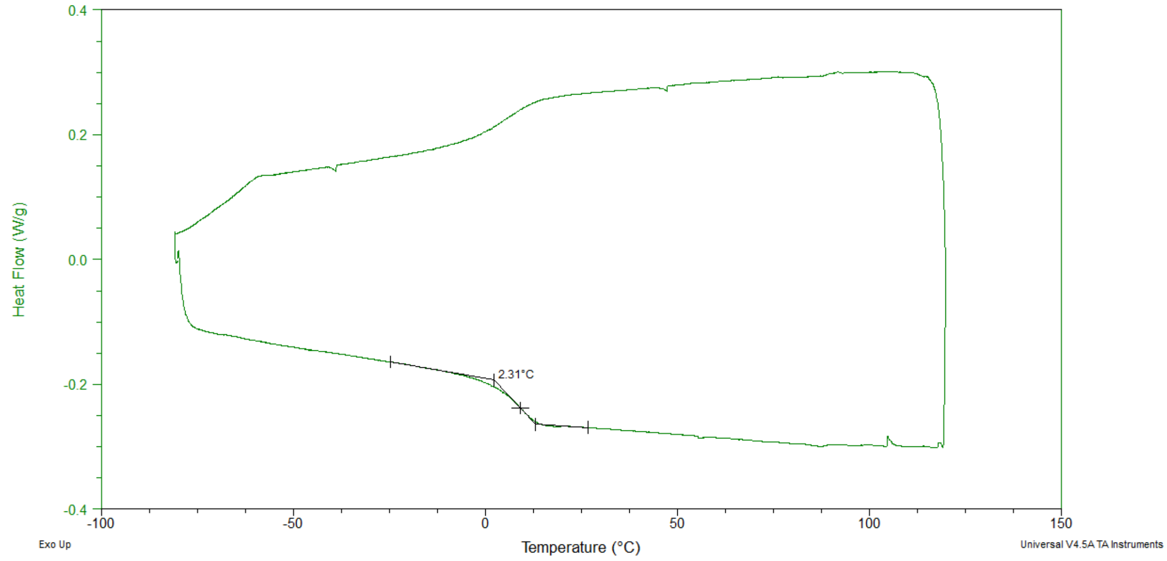

Figure S10. An example DSC Thermogram for the AN07 elastomer, showing the second heat/cool cycle, with exothermic transitions orientated upwards.

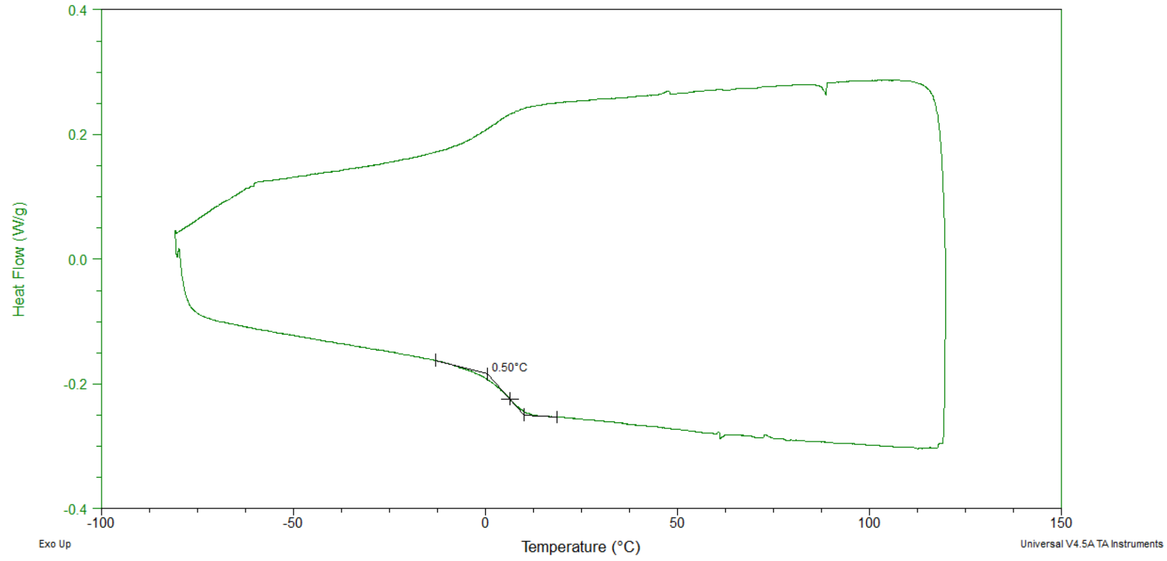

Figure S11. An example DSC Thermogram for the AN08 elastomer, showing the second heat/cool cycle, with exothermic transitions orientated upwards.

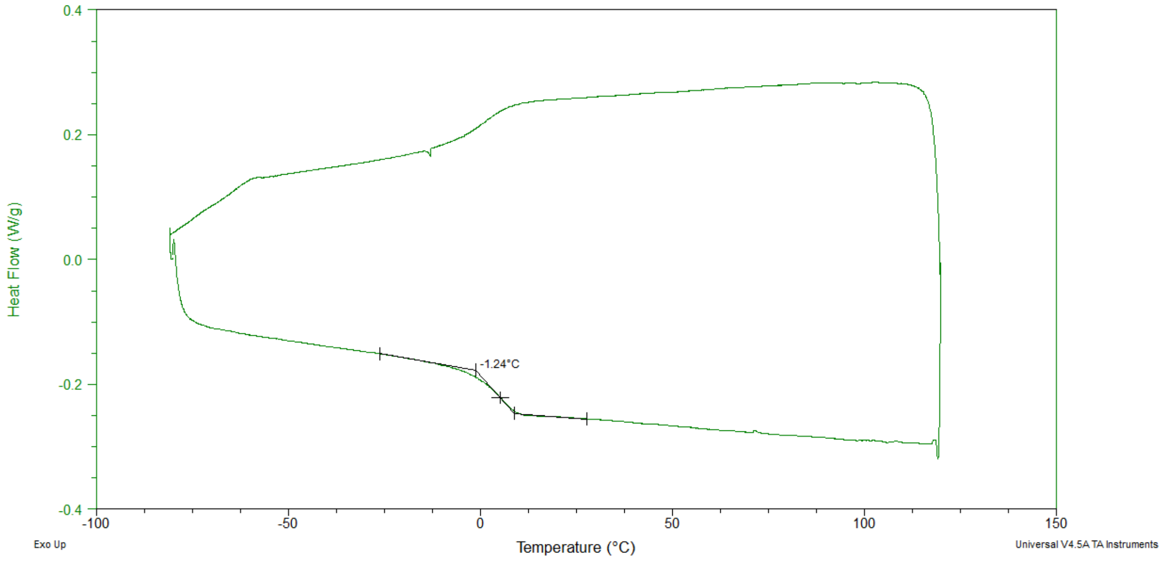

Figure S12. An example DSC Thermogram for the AN09 elastomer, showing the second heat/cool cycle, with exothermic transitions orientated upwards.

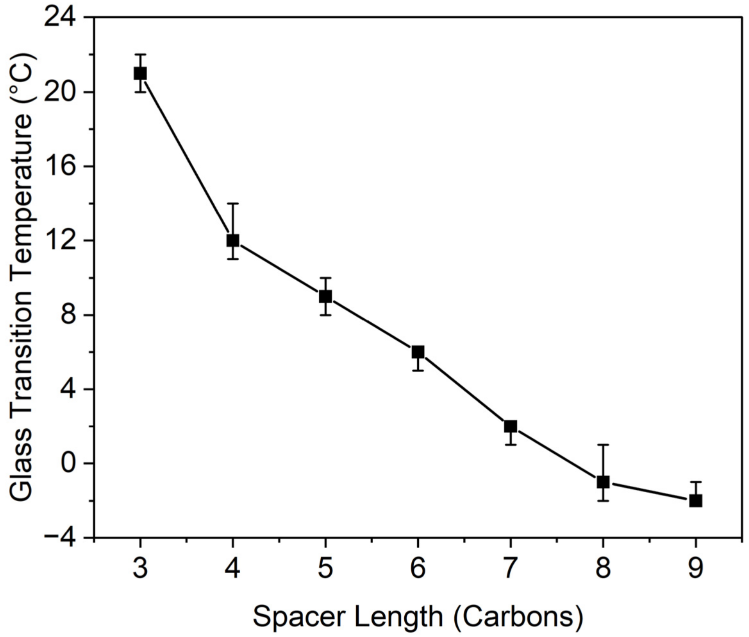

Figure S13 . The effect of spacer length on the glass transition temperature of the LCEs. In all cases error bars indicate the range of values recorded.

Thermally Induced Shape Change Of LCEs

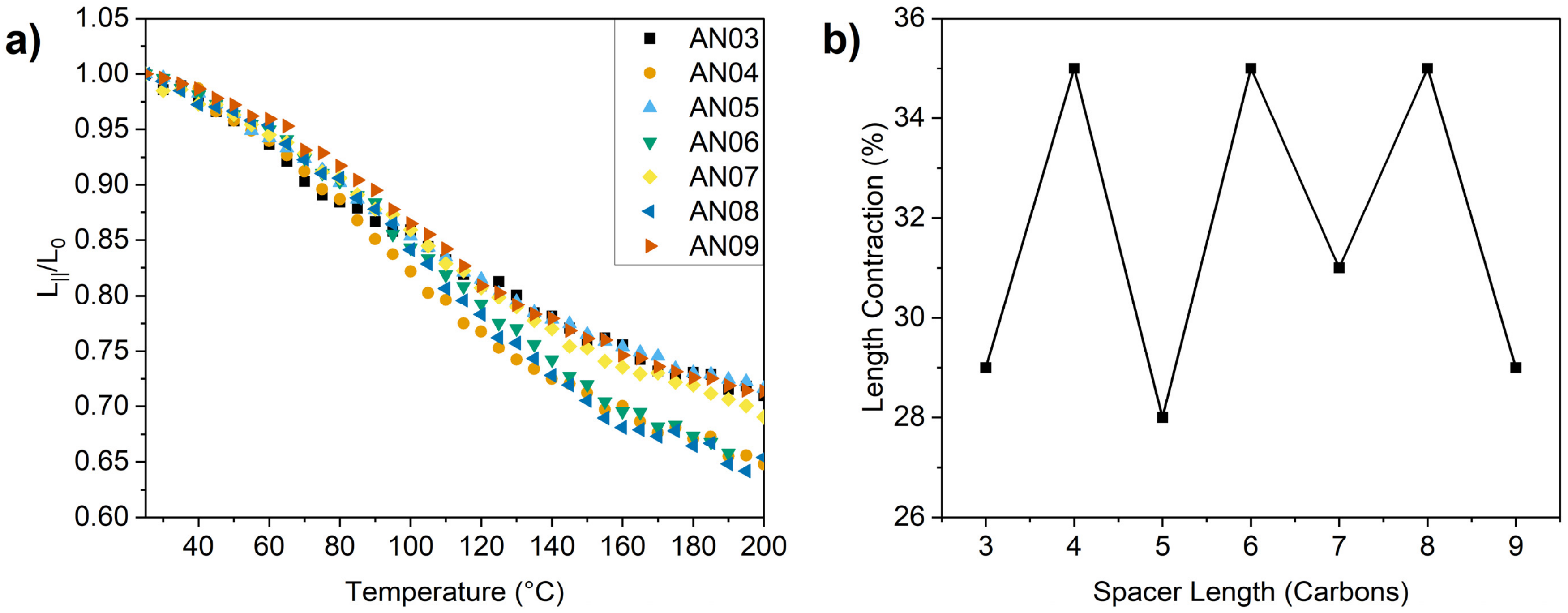

Figure S14. a) Length contraction parallel to the director as a function of temperature, for the LCEs of varying spacer length; b) The length contraction of the LCEs parallel to the director at 200 °C as a function of spacer length.

Order Parameter Measurements Of LCEs

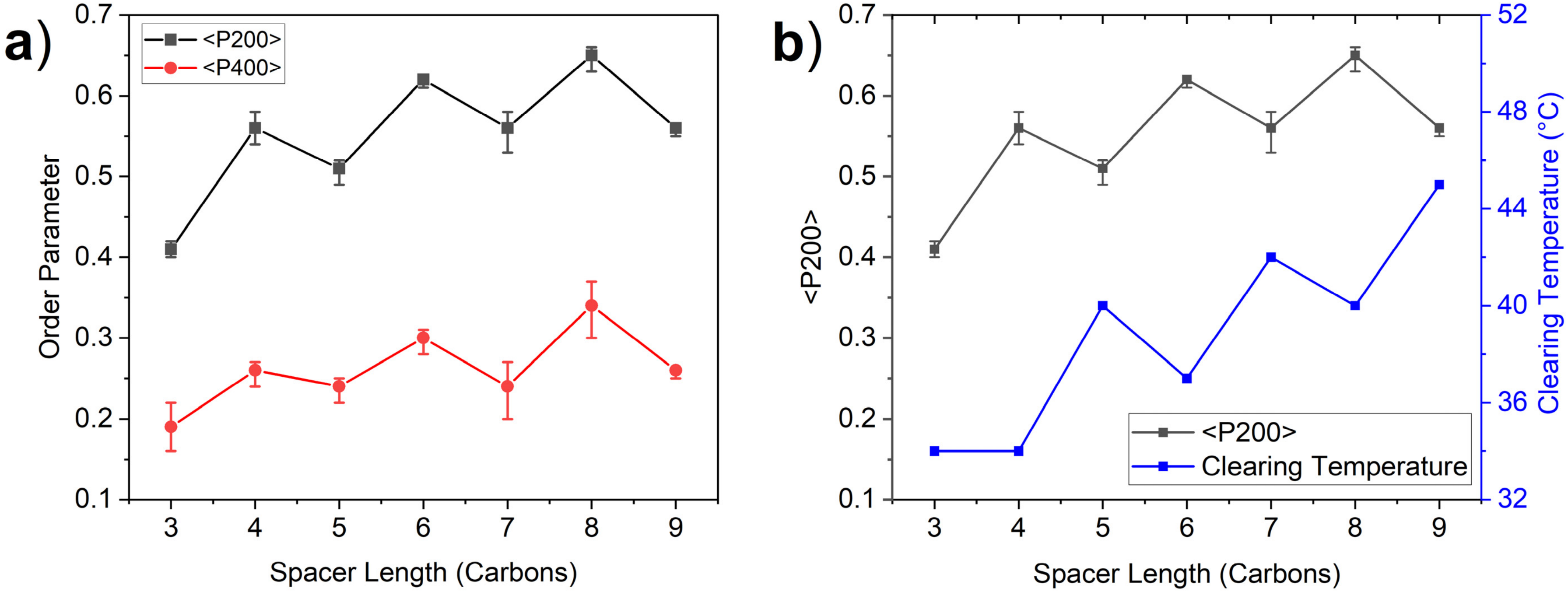

Figure S15. a) The <P200> and <P400> order parameters of the LCEs as a function of spacer length, and b) the <P200> order parameter of the LCEs and the clearing temperature of the uncured precursor mixtures, as a function of spacer length. In both cases, error bars denote the range of values obtained across multiple samples.

Variable Temperature Small Angle X-Ray Scattering

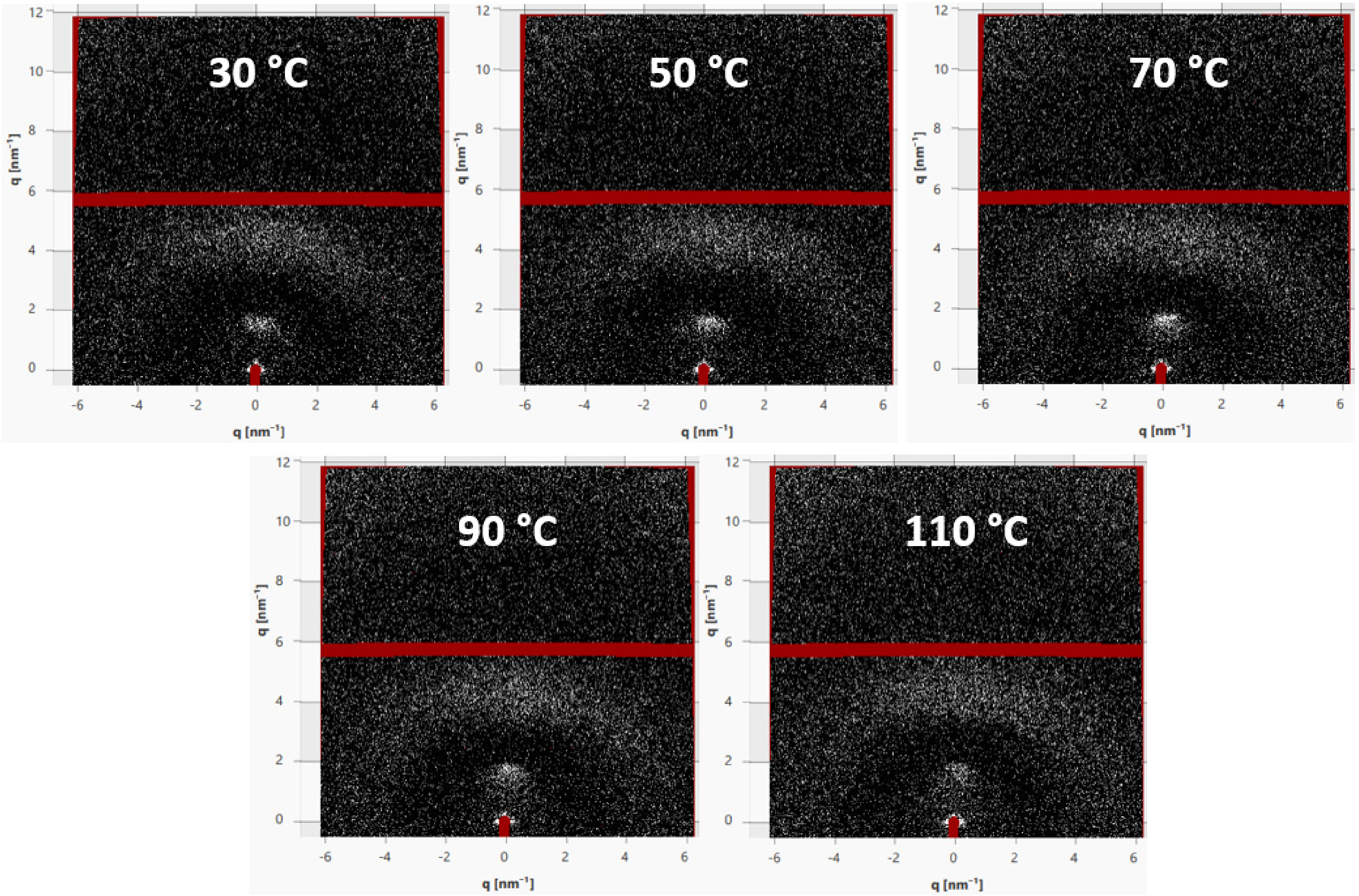

Figure S16. 2D SAXS data as a function of temperature for the AN07 LCE.

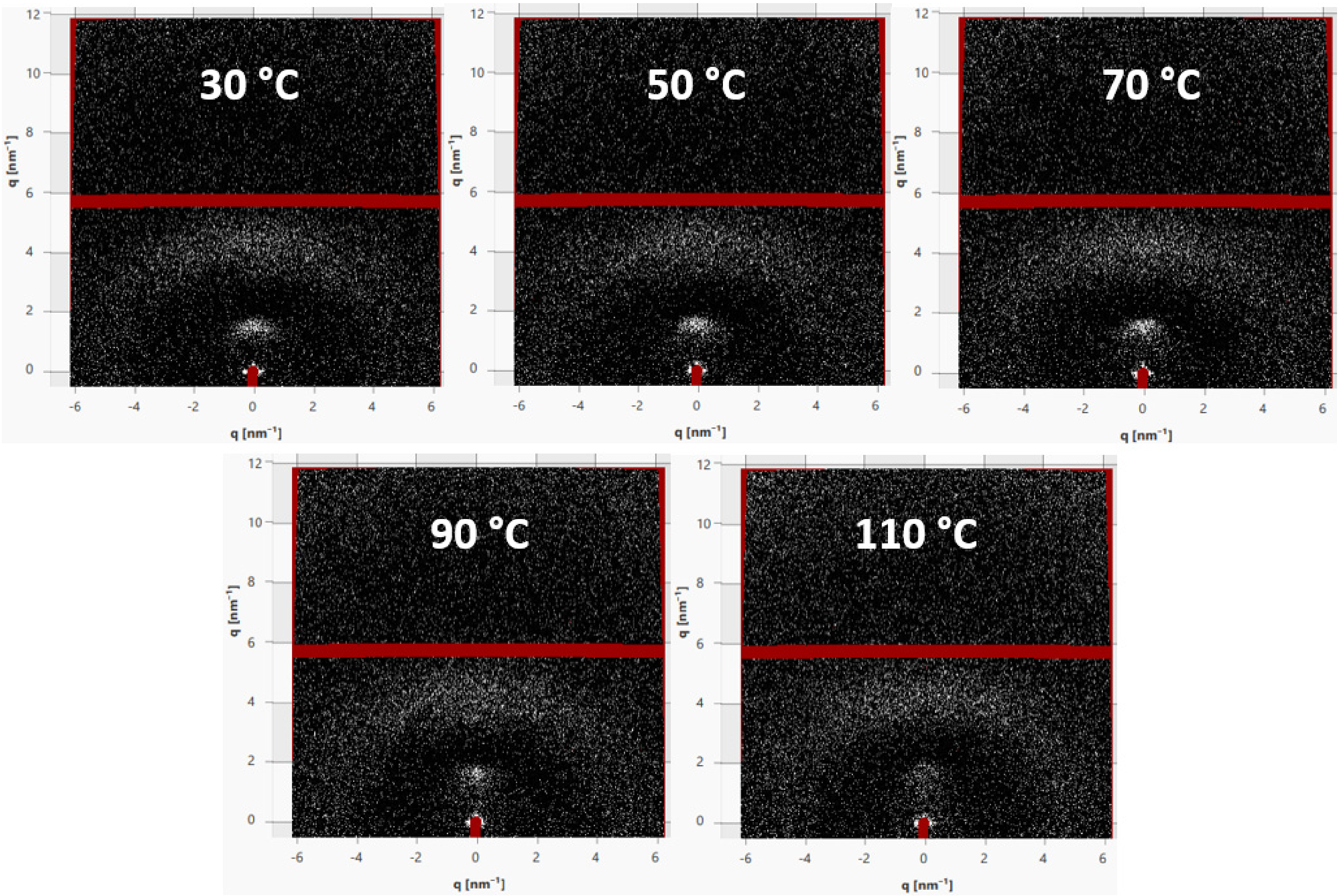

Figure S17. 2D SAXS data as a function of temperature for the AN08 LCE.

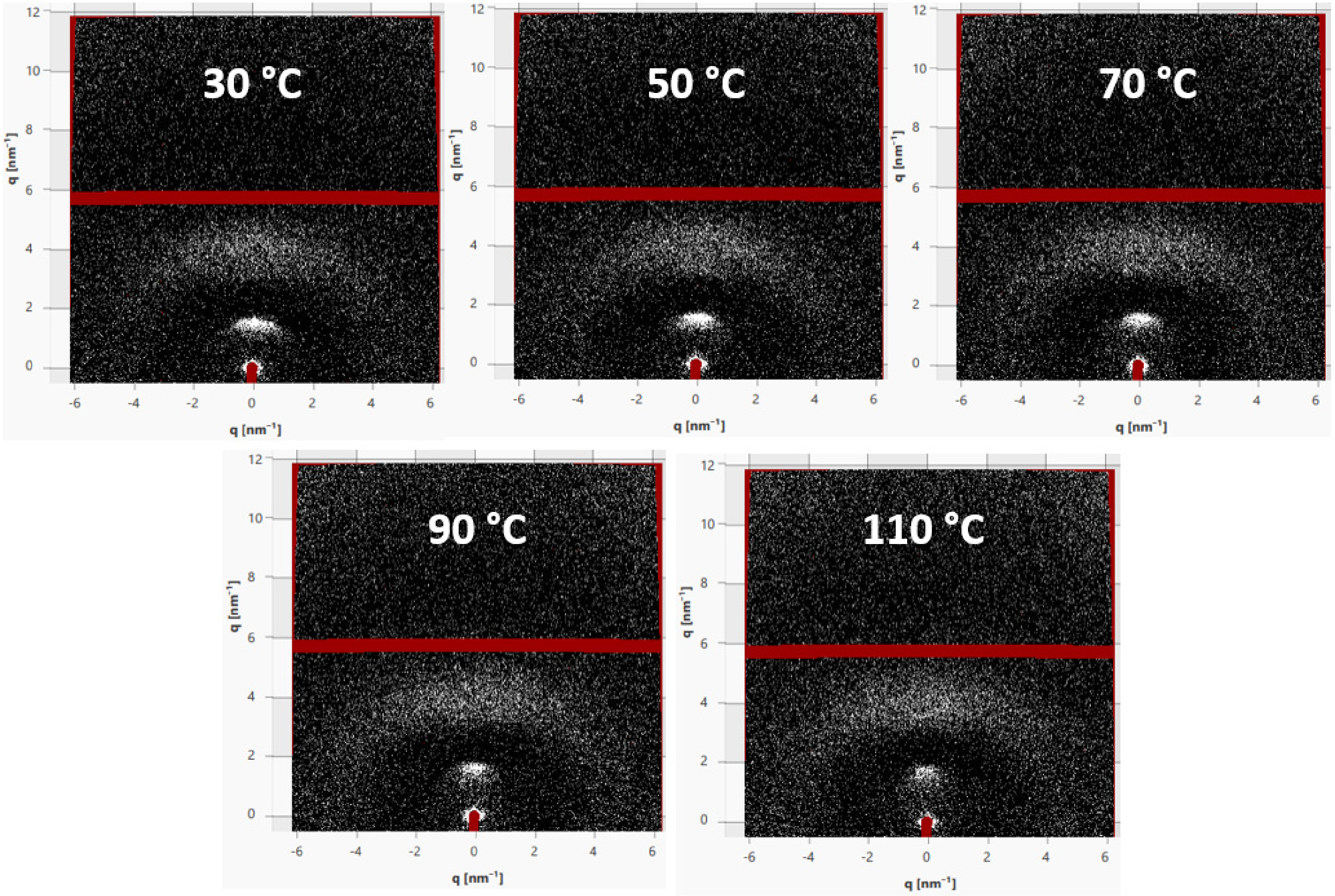

Figure S18. 2D SAXS data as a function of temperature for the AN09 LCE.

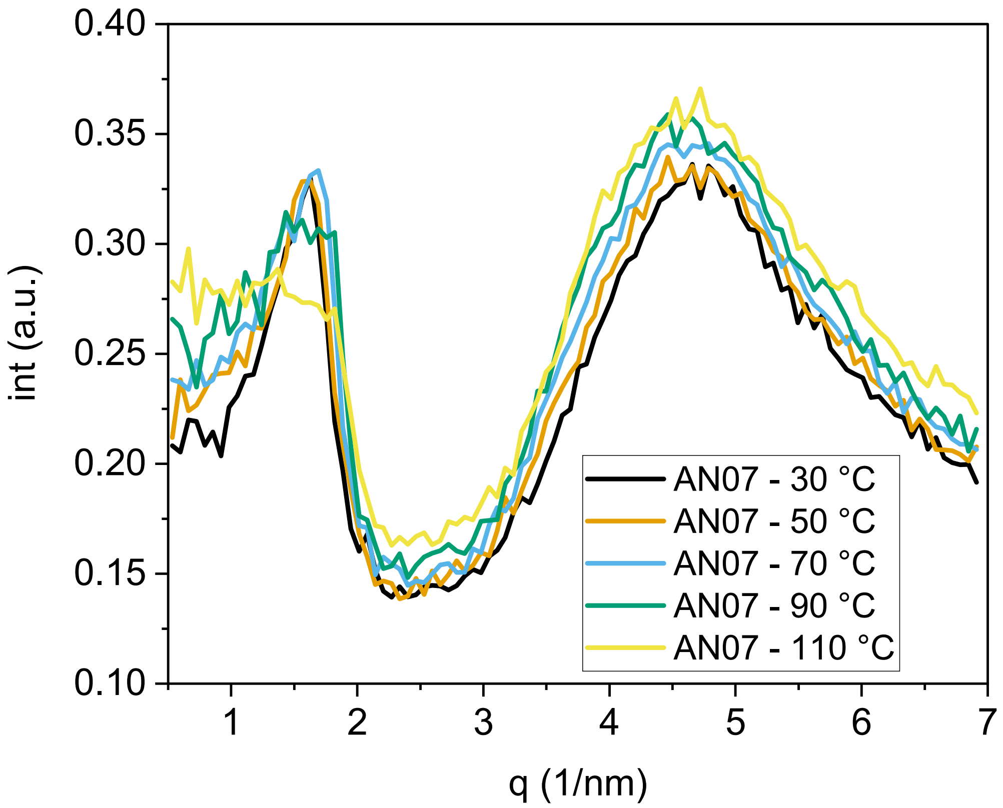

Figure S19. 1D SAXS data as a function of temperature for the AN07 LCE.

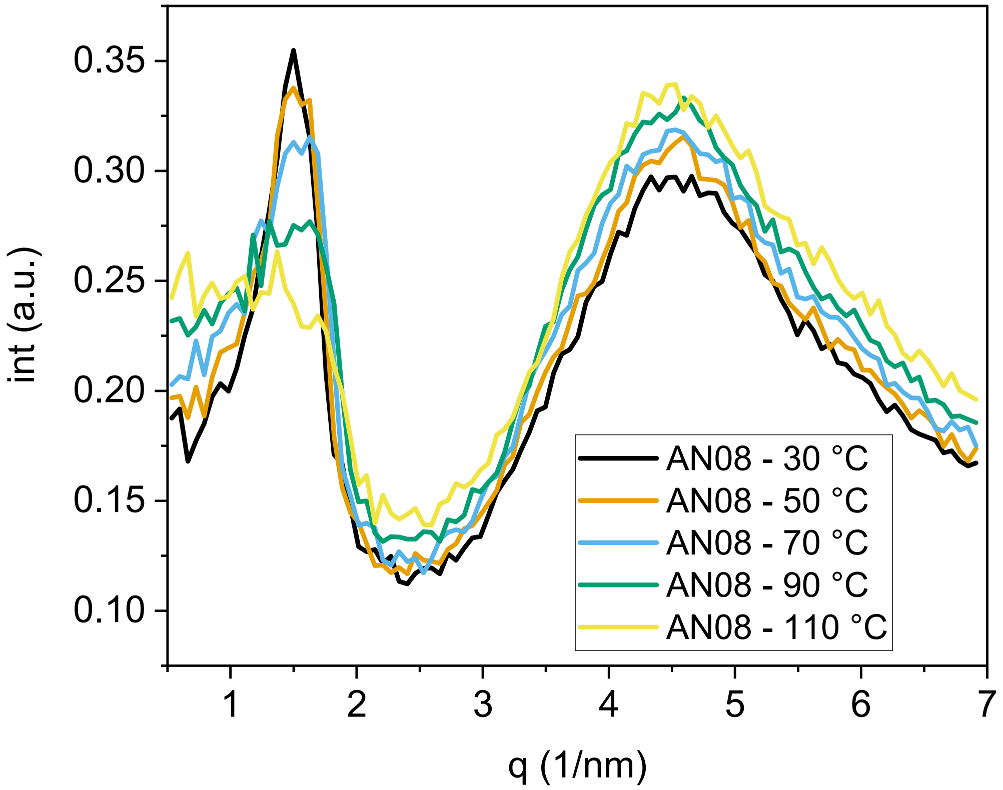

Figure S20. 1D SAXS data as a function of temperature for the AN08 LCE

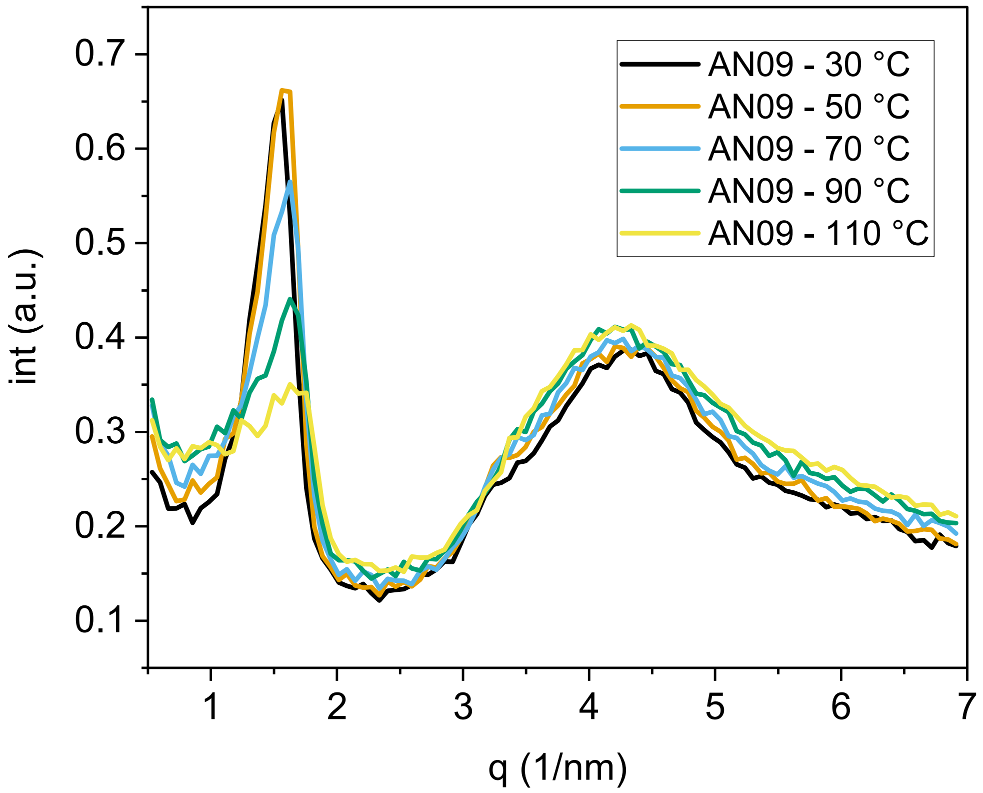

Figure S21. 1D SAXS data as a function of temperature for the AN09 LCE.

References

(1) Wang, Z.; Raistrick, T.; Street, A.; Reynolds, M.; Liu, Y.; Gleeson, H. F. Direct Observation of Biaxial Nematic Order in Auxetic Liquid Crystal Elastomers. *Materials* **2023**, *16* (1), 393. https://doi.org/10.3390/ma16010393.

(2) Raistrick, T.; Zhang, Z.; Mistry, D.; Mattsson, J.; Gleeson, H. F. Understanding the Physics of the Auxetic Response in a Liquid Crystal Elastomer. *Phys Rev Res* **2021**, *3* (2), 023191. https://doi.org/10.1103/PhysRevResearch.3.023191.

(3) Mistry, D.; Connell, S. D.; Mickthwaite, S. L.; Morgan, P. B.; Clamp, J. H.; Gleeson, H. F. Coincident Molecular Auxeticity and Negative Order Parameter in a Liquid Crystal Elastomer. *Nat Commun* **2018**, *9* (1), 5095. https://doi.org/10.1038/s41467-018-07587-y.

(4) Dunning, T. H. Gaussian Basis Sets for Use in Correlated Molecular Calculations. I. The Atoms Boron through Neon and Hydrogen. *J Chem Phys* **1989**, *90* (2), 1007–1023. https://doi.org/10.1063/1.456153.

(5) Becke, A. D. Density-Functional Thermochemistry. III. The Role of Exact Exchange. *J Chem Phys* **1993**, *98* (7), 5648–5652. https://doi.org/10.1063/1.464913.
